# Investigating the potential anti-depressive mechanisms of statins: a transcriptomic and Mendelian randomization analysis

**DOI:** 10.1038/s41398-023-02403-8

**Published:** 2023-04-04

**Authors:** Jiayue-Clara Jiang, Chenwen Hu, Andrew M. McIntosh, Sonia Shah

**Affiliations:** 1 Institute for Molecular Bioscience, The University of Queensland, St Lucia, QLD Australia; 2 The University of Queensland, St Lucia, QLD Australia; 3 Division of Psychiatry, University of Edinburgh, Edinburgh, UK

**Keywords:** Genomics, Pharmacogenomics

## Abstract

Observational studies and randomized controlled trials presented inconsistent findings on the effects of cholesterol-lowering statins on depression. It therefore remains unclear whether statins have any beneficial effects on depression, and if so, what the underlying molecular mechanisms are. Here, we aimed to use genomic approaches to investigate this further. Using Connectivity Map (CMap), we first investigated whether statins and antidepressants shared pharmacological effects by interrogating gene expression responses to drug exposure in human cell lines. Second, using Mendelian randomization analysis, we investigated both on-target (through HMGCR inhibition) and potential off-target (through ITGAL and HDAC2 inhibition) causal effects of statins on depression risk and depressive symptoms, and traits related to the shared biological pathways identified from CMap analysis. Compounds inducing highly similar gene expression responses to statins in HA1E cells (indicated by an average connectivity score with statins > 90) were found to be enriched for antidepressants (12 out of 38 antidepressants; *p* = 9E-08). Genes perturbed in the same direction by both statins and antidepressants were significantly enriched for diverse cellular and metabolic pathways, and various immune activation, development and response processes. MR analysis did not identify any significant associations between statin exposure and depression risk or symptoms after multiple testing correction. However, genetically proxied HMGCR inhibition was strongly associated with alterations in platelets (a prominent serotonin reservoir) and monocyte percentage, which have previously been implicated in depression. Genetically proxied ITGAL inhibition was strongly associated with basophil, monocyte and neutrophil counts. We identified biological pathways that are commonly perturbed by both statins and antidepressants, and haematological biomarkers genetically associated with statin targets. Our findings warrant pre-clinical investigation of the causal role of these shared pathways in depression and potential as therapeutic targets, and investigation of whether blood biomarkers may be important considerations in clinical trials investigating effects of statins on depression.

## Introduction

Depression is one of the most common mental disorders and a leading cause of non-fatal health loss [1]. Pharmacological treatments for depression involve the use of antidepressants to alleviate symptoms. However, conventional antidepressants often show delayed treatment response and limited remission rates [2], warranting the need for new treatments.

Drug repurposing is the process of identifying novel therapeutic effects of existing drugs [3]. Since the safety profile of approved drugs is often well-established, drug repurposing presents an efficient and cost-effective approach for developing new pharmacological treatments for depression. One such class of drugs that have gained some interest in this regard are statins. Statins inhibit 3-Hydroxy-3-Methylglutaryl Coenzyme A Reductase (HMGCR), effectively lowering low-density lipoprotein cholesterol (LDL-C) levels [4], and are widely prescribed for the prevention and management of cardiovascular diseases [5]. While small-scaled randomized controlled trials (RCT) have reported clinical benefits of statins on depressive symptoms when used as an add-on treatment to antidepressants [6], there is overall conflicting evidence on the anti-depressive effect of statins from observational studies and randomized controlled trials [6–13]. This inconsistency in findings may be due to differences in the chemical and pharmacokinetic properties of statin compounds studied, small sample sizes, varying follow-up times, unmeasured confounders and heterogeneity in depression pathophysiology [6]. Despite inconsistent evidence on the causal relationship, in vivo, animal and human studies have proposed several mechanisms to explain the potential anti-depressive effects of statins [14]. This includes direct impact on the central nervous system processes, such as antidepressant-like modulation of serotonin levels in the hippocampus, activation of neuroprotective pathways, protection against neurotoxicity and increased neurogenesis [14]. In addition, statins have been found to reduce thrombogenesis and increase cerebral blood flow, a process linked to depression biology [14]. Most notably, statins have demonstrated diverse immunomodulatory and anti-inflammatory effects (both dependent and independent of their lipid-lowering activity), such as downregulation of C-reactive protein (CRP), interleukins and tumour necrosis factor (TNF), many of which have been implicated in depression risk [14]. However, any beneficial effect of statins on depression and how such effects may be mediated still remain unclear.

Analysis of large genomic datasets can provide an alternative approach to investigating the mechanisms of action and repurposing potential of drugs. Genome-wide association studies (GWAS) have guided drug repurposing strategies for several diseases, with a notable example being the repurposing of a psoriasis treatment, Ustekinumab, for Crohn’s disease [3, 15]. Mendelian randomization (MR) is a statistical genomics approach that uses genetic proxies for an exposure of interest to investigate its causal effect on an outcome, essentially mimicking a RCT [16]. With genetic studies nowadays often including hundreds of thousands of individuals, MR provides a powerful approach to overcome some of the caveats of observational studies, such as unmeasured confounders, inability to infer causality and sample size issues [16]. An example of this is the contradictory findings between observational studies and RCTs regarding the association between high-density lipoprotein cholesterol (HDL-C) and risk of myocardial infarction (MI). Despite positive findings from observational studies, MR analysis showed no causal association between genetic modulation of HDL-C levels and MI risk, which is concordant with the findings from multiple RCTs that failed to report consistent clinical benefits of HDL-C-raising agents on reducing MI risk [17]. Another computational approach is signature matching, where the molecular changes (e.g. gene or protein expression) in response to drug candidate exposure are compared against drugs approved for treating the disease. The underlying assumption is that if two drugs have shared pharmacological effects, they likely induce highly similar molecular responses [3]. The Connectivity Map (CMap) is a database of drug gene expression signatures that can be used for signature matching analysis [18], and an example of this is the use of CMap data in the development of novel treatments for obesity [19].

In this study we aimed to use genomic approaches to investigate the repurposing potential of statins for the treatment of depression. More specifically, we applied both the signature matching approach (using CMap data) and MR analysis to investigate whether exposure to statins may have any effects on depression, and identify molecular pathways through which any effects on depression may be mediated.

## Methods

All R analyses described in this study were performed in R version 4.1.0 unless otherwise stated. Further details are presented in Supplementary Methods, Supplementary Figures and Supplementary Tables contained in Additional File 1.

### CMap gene expression signatures

CMap gene expression profiles comprise of 978 directly measured genes (hereby referred to as “landmark” genes) and 11,350 genes whose expressions have been imputed (referred to as “inferred” genes) (Supplementary Methods) [18]. The gene expression signature of a compound is defined by the gene expression changes (presented as z-scores) of 12,328 (landmark and inferred) genes, induced by compound exposure in a human cell line [18].

### Connectivity scores

The CMap platform computes a “connectivity” score (Tau) as a measure of similarity between a query gene expression signature, in this case the signature for statin exposure, and a reference signature (here we are interested in signatures of antidepressants) (Supplementary Methods and Supplementary Fig. 1) [18]. A subset of the CMap database, termed the “Touchstone” collection, contains gene expression signatures for well-annotated chemical compounds, as well as gene knock-down or overexpression studies (referred to as genetic perturbations), profiled in a “core” set consisting of nine cancer cell lines (A375, A549, HA1E, HCC515, HEPG2, HT29, MCF7, PC3 and VCAP). By comparing the query signature against the Touchstone dataset on the CLUE platform (https://clue.io), CMap generates nine cell-type-specific connectivity profiles, one from each core cell line, and computes a summary connectivity profile, which is summarized across the nine cell lines as described in Subramanian et al. [18] (Supplementary Methods and Supplementary Fig. 1).

The Tau scores range between −100 and 100, and the magnitude of the score corresponds to the magnitude of similarity or dissimilarity between the query and reference gene expression signatures. In general, a Tau score greater than 90 is used as a threshold for identifying compounds with potentially shared pharmacological effects [18]. By default, CMap constructs a query signature for each compound using the 50 most upregulated and 50 most downregulated landmark genes to calculate the Tau scores with other compounds in the CMap database.

### Statin connectivity profiles summarized across cell lines

As a preliminary analysis, we first investigated the connectivity profile of statin compounds summarized across the nine core cell lines, by querying the connectivity profiles of “HMGCR inhibitors” using the online CLUE platform (https://clue.io) [18].

### Analysis of gene expression signatures in HA1E cells

To identify the biological processes perturbed by statin and antidepressant exposure, we retrieved the cell-type-specific CMap gene expression signatures from the GEO repository (GSE92742) (accessed on 8 July 2021). The most comprehensive gene expression signatures are available for nine core cancer cell lines (Supplementary Methods). We selected the signatures of statins in the HA1E kidney cell line for analysis, due to the greatest concordance and specificity in gene expression changes between different statin compounds (Supplementary Fig. 2 and Supplementary Methods). In total, we selected the signatures of seven statins (atorvastatin, fluvastatin, lovastatin, mevastatin, pravastatin, rosuvastatin and simvastatin) in HA1E cells (after 24 h of exposure at a drug concentration of 10 μM), with pravastatin discarded from further analysis due to low correlation with other statins (Supplementary Fig. 3). We also retrieved the gene expression signatures of antidepressants (38 antidepressants in total were profiled in HA1E cells), and included alvespimycin (heat shock protein inhibitor) and sirolimus (mTOR inhibitor) as control compounds with no known anti-depressive effects. While limited evidence has suggested a cholesterol-modifying effect of sirolimus [20, 21], the effect of alvespimycin on lipids remains unstudied. The CMap signatures analyzed are summarized in Supplementary Table 1.

### Pathway enrichment analysis for genes perturbed by statins in HA1E cells

For statin gene signatures generated in HA1E cells, we performed pathway enrichment analysis on genes upregulated and downregulated in response to statin exposure, defined using z-score > 1 and z-score < −1 respectively. Although there is no consensus on the z-score threshold for identifying perturbed pathways, it has been demonstrated that for most compounds at the profiled concentration in CMap, changes in gene expression are subtle [22]. We therefore applied a threshold of |z| > 1 for the primary analysis, and used more stringent thresholds (|z| of 1.5 and 2) in sensitivity analysis (Supplementary Table 2).

To identify Gene Ontology (GO) biological processes perturbed by statins, functional enrichment analysis by gProfiler2 (version 0.2.0; Ensembl 104) was performed for upregulated and downregulated genes separately in R [23, 24], using all 12,328 CMap-profiled genes as the background gene list and a gSCS-corrected *p*-value < 0.05 to indicate statistical significance. The gSCS multiple testing correction method accounts for the overlapping and hierarchically related nature of GO functional terms and gives a more stringent significance threshold [23, 24]. Given the many overlapping GO terms, we categorized the significantly enriched biological process terms into higher-level ancestor terms (Supplementary Methods).

### Statin connectivity profiles in HA1E cells

Using statin signatures from HA1E cells, we computed the HA1E-specific connectivity profile, in order to investigate their similarity to the signatures induced by other compounds in the same cell line (Supplementary Fig. 1). For each statin, we constructed a query signature using 50 most upregulated and 50 most downregulated landmark genes defined by z-scores (referred to as the top 50 gene set), which is the default criteria used by CMap to generate Tau scores. As a sensitivity analysis, we re-computed the connectivity profiles for statins using the top 100 and 150 upregulated and downregulated landmark genes from their gene expression signatures. The query signature for each statin was compared against the Touchstone reference signatures in the CMap database and Tau scores were retrieved using the CLUE platform. If the same compound was profiled more than once (indicated by more than one CMap compound ID), we retained only the compound ID profiled in the Touchstone collection. The similarity between specific gene expression signatures was further evaluated by investigating the Pearson correlation of the z-scores of all 12,328 genes profiled in each CMap signature (Supplementary Methods).

### Enrichment of antidepressants amongst high-connectivity compounds

The HA1E-specific statin connectivity profiles contained the connectivity scores between the statins and a total of 38 antidepressants (ATC code: N06A) (Supplementary Table 3 and Supplementary Methods). We performed a chi-square test to determine enrichment of antidepressants among chemical compounds with high positive connectivity with statins (average Tau > 90). A chi-square *p*-value < 0.05 defined statistical significance.

### Pathway enrichment analysis for genes perturbed by both statins and antidepressants in HA1E cells

Out of the antidepressants with highly similar gene expression signatures to statins in HA1E cells, we selected the top five antidepressants that demonstrated the highest average connectivity with the six statins for detailed analysis, namely three tricyclic antidepressants (desipramine, trimipramine and nortriptyline) and two selective serotonin reuptake inhibitors (paroxetine and sertraline) (Additional File 2). For each statin-antidepressant pair, we identified genes that were differentially expressed (absolute z > 1) in response to both compounds, and performed pathway enrichment analysis on genes perturbed in the same and opposite direction (Supplementary Methods).

### Replication of CMap analysis in NPC cells

We also analyzed the CMap gene expression signatures of statins in neural progenitor cells (NPC) as a sensitivity analysis. It is important to note that although NPC cells are precursors of glial and neuronal cells, and thus biologically more relevant to depression, they are less well understood than the HA1E cell line, and consist of a heterogenous population of cells with diverse lineage commitments [25, 26] (Supplementary Methods). Unlike the nine core cancer cell lines, the gene expression signatures of a reduced number of chemical compounds, under a limited variety of exposure conditions, have been profiled in the NPC cell line by CMap [18].

As described above, we identified biological processes perturbed by statins by performing functional enrichment analysis on genes upregulated and downregulated by statins in NPC cells, defined using |z| > 1, and performed sensitivity analysis using more stringent z-score thresholds (|z| of 1.5 and 2).

We computed statin connectivity profiles using the 50 most upregulated and 50 most downregulated landmark genes from the statin gene expression signatures in NPC cells. We selected the summary connectivity score profiles for analysis, which were summarized across the nine core cancer cell lines profiled by CMap, as an NPC-specific connectivity profile was not available (details described in Supplementary Methods and Supplementary Fig. 1). The connectivity scores of a total of 38 antidepressants were available in the summary connectivity profiles, and similar to the analysis of HA1E cells, we performed a chi-square test to test for the enrichment of antidepressants amongst chemical compounds that showed high summary connectivity scores (Tau > 90) with the statin query signatures generated from NPC cells. For each statin-antidepressant pairs investigated in HA1E cells, we replicated the functional enrichment analysis of common differentially expressed genes using the gene expression signatures of statins and antidepressants in NPC cells.

### Mendelian randomization

MR is a statistical genetics approach that uses genetic variants associated with the exposure of interest, in this case statin exposure, to provide an unbiased estimate of the causal effect of an exposure on an outcome of interest (Supplementary Methods). Statins are potent inhibitors of the HMGCR protein, and statin-mediated HMGCR inhibition is the primary pathway underlying their lipid-lowering effects [27]. We queried DrugBank [28] for any known off-target effects of statins, and found that selective statins have been reported to exert in vitro off-target inhibition of Integrin Alpha-L (ITGAL) and Histone Deacetylase 2 (HDAC2) (Supplementary Methods), though this off-target inhibition is not related to the cholesterol-lowering properties of statins [29–32]. We investigated both on-target and off-target effects of statins using MR.

### Genetic instruments for statin target inhibition

The most effective genetic instrument for statin exposure would be a loss-of-function variant in the *HMGCR* gene. However, such variants are rare and not always feasible with currently available genetic datasets [33]. Alternatively, common genetic variants associated with drug target gene expression can be used as instruments [34]. We used expression quantitative trait loci (eQTL) of *HMGCR*, *ITGAL* and *HDAC2* expression in blood from the largest available eQTL dataset (eQTLGen data; *N* = 31,684 [35]) to proxy for statin exposure (Supplementary Methods and Supplementary Table 4). Out of the eQTLs that were also profiled across the outcome GWAS datasets, we selected previously validated eQTL instruments, or the strongest eQTL for each gene as the genetic instrument. We used eQTLs with F-statistic > 10, indicating strong instruments. To investigate the on-target effect of statins, we selected rs12916, which is a previously validated instrument for HMGCR inhibition [36], and a strong cis-eQTL for *HMGCR* expression in blood (p = 1.5E-36). We performed additional analysis using a strong eQTL (rs17671591; *p* = 2.5E-05) for *HMGCR* expression in the brain prefrontal cortex (PsychENCODE [37]), which is in moderate linkage disequilibrium (LD) (r^2^ = 0.6) with rs12916 amongst European-ancestry individuals. To make inferences about the off-target effects of statins, we used the most significant blood eQTLs as genetic proxies for ITGAL (rs11574938; *p* = 7.9E-150) and HDAC2 (rs9481408; *p* = 4.1E-07) inhibition.

### Outcome GWAS data

Publicly available GWAS summary statistics, derived from European-ancestry cohorts, were obtained for 29 depression-related and immune traits, including depression risk, depression-related symptoms (neuroticism, worrying and depressed affect), haematological traits, interleukin-6 (IL6) and CRP levels. In addition, we included several proof-of-principle traits previously linked to statin use, including blood lipid levels (HDL-C, LDL-C, and triglycerides (TG)), body mass index (BMI), coronary artery disease (CAD) and type II diabetes (T2D). Details of each GWAS are provided in Supplementary Table 5.

### SMR analysis

We performed MR analysis using Summary-based Mendelian Randomization (SMR) [38] (version 0.710) to evaluate the association of genetically proxied statin on-target and off-target inhibition with various traits. We used blood (*HMGCR*, *ITGAL* and *HDAC2*) or brain (*HMGCR*) gene expression as exposure, and disease and haematological traits as outcome. GWAS summary-data-based MR methods, such as SMR, require a reference dataset for LD estimation, and we used a random sample of 10,000 European-ancestry individuals from the UK Biobank as the LD reference [39]. A threshold of *p* < 0.00057 (after multiple testing correction for three statin target genes and 29 traits of interest) defined statistical significance for the SMR test. We performed the heterogeneity in dependent instruments (HEIDI) test as a sensitivity analysis [38], where a HEIDI *p*-value > 0.01 indicated that the observed exposure-outcome association was mediated through one causal single nucleotide polymorphism (SNP), one of the assumptions of MR, rather than via LD between separate SNPs (linkage scenario) (Supplementary Methods).

## Results

### Statins induce gene expression changes in lipid and immune-associated pathways

As would be expected for drugs with the same mechanism of action, we observed a high correlation in the gene expression signatures across six statins (atorvastatin, fluvastatin, lovastatin, mevastatin, rosuvastatin, and simvastatin) in HA1E cells (Supplementary Fig. 4), supported by high pairwise connectivity scores (median Tau = 99.95).

Functional enrichment analysis identified a total of 366 GO biological process terms that were significantly enriched amongst the upregulated (z-score > 1) and downregulated (z-score < −1) genes induced by at least one statin in HA1E cells (Supplementary Fig. 5 and Additional File 3). Cellular processes, including various DNA replication and RNA processing pathways, were the most enriched category of biological processes (Supplementary Fig. 5). Statins also induced extensive perturbations in metabolism, biological regulation, localization, response to stimulus and immune processes. As expected from their lipid-lowering effects, statins induced widespread expression changes amongst genes annotated with lipid metabolic pathways. We confirmed the reproducibility of pathway enrichment analysis results using more stringent thresholds for defining differentially expressed genes (|z| > 1.5 or 2) (Supplementary Fig. 6).

### Compounds with high connectivity to statins are enriched for antidepressants

**Fig. 1:**
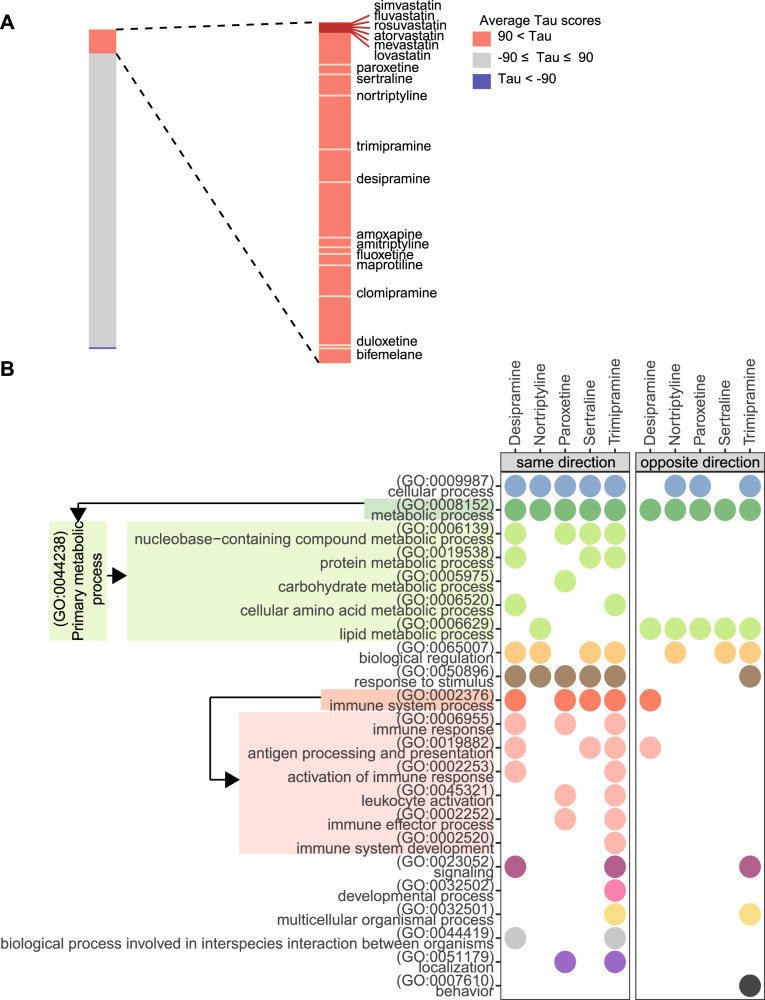
Statins and antidepressants induced similar transcriptomic responses in HA1E cells. **A** Statin and antidepressant reference signatures ranked by average connectivity (Tau) scores to the six statin query signatures. Lengths of the coloured bars represent the number of compounds with the corresponding average Tau scores (red: Tau > 90; grey: −90 ≤ Tau ≤ 90; blue: Tau < −90). Antidepressants with an average Tau score higher than 90 are shown. **B** Biological processes identified as significantly enriched among genes perturbed by both statins and antidepressants, in the same (left panel) and opposite (right panel) directions. *Y*-axis shows the ancestor GO biological process terms, as well as the child terms of primary metabolic process (GO:0044238) (a child term of “metabolic process”) and immune system process (GO:0002376). Arrows indicate ancestor-to-child relationships between GO terms. The bubble plots show the antidepressants for which the biological processes were identified as significantly enriched (gSCS-corrected *p*-value < 0.05) in at least one statin-antidepressant pair.

In the preliminary analysis, using connectivity scores summarized across the nine core cell lines, we identified several antidepressant classes, namely tricyclic antidepressants and norepinephrine reuptake inhibitors, amongst the top compound classes that induced highly similar gene expression changes to statins (Supplementary Fig. 7). Comprehensive analysis of statin gene expression signatures in HA1E cells identified 182 out of 2484 (7.3%) non-statin compounds with an average Tau score greater than 90, indicating strong positive correlation to statin-induced gene expression changes (Additional File 2). Twelve compounds had a Tau score lower than −90, suggesting opposite transcriptomic impacts (Additional File 4). Antidepressants were significantly enriched amongst compounds with highly similar gene expression signatures to statins (*p* = 9E-08) (Supplementary Table 6), with 12 out of the 38 (31.6%) antidepressants profiled by the CMap database showing an average Tau score greater than 90 across the six statins (Fig. 1A). The enrichment of antidepressants amongst high-connectivity compounds remained significant when we re-computed the connectivity profile for statins using the top 100 and 150 most differentially expressed landmark genes (Supplementary Table 6).

### Immune pathways are perturbed by statins and antidepressants in concordant direction in HA1E cells

Functional enrichment analysis was performed on genes perturbed by both statins and antidepressants that showed high connectivity (desipramine, trimipramine, nortriptyline, paroxetine and sertraline) (Fig. 1A, Supplementary Fig. 8 and Supplementary Fig. 9), and identified 179 and 56 biological processes to be significantly enriched in genes perturbed in the same and opposite directions, respectively, by at least one statin-antidepressant pair (Additional File 5). Notably, lipid metabolic processes were mostly enriched amongst genes perturbed in the opposite direction, supporting previous findings on the cholesterol-increasing effects of antidepressants (Fig. 1B) [40]. In comparison, genes perturbed in the same direction were, to a higher extent, functionally involved in cellular processes and biological regulation processes, such as cell cycle regulation, RNA processing, and cellular component biogenesis (Additional File 5). Furthermore, genes perturbed in the same direction were enriched in diverse immune system processes involved in both innate and adaptive immunity, such as granulocyte activation, myeloid leucocyte activation, T-cell receptor signalling, as well as antigen processing and presentation (Additional File 5). Pathways involving inflammatory cytokines, such as interleukin-1 and TNF, were also found to be perturbed in the same direction, suggesting a shared pharmacological impact of statins and antidepressants on the inflammatory pathways (Additional File 5). Widespread enrichment of immune pathways was not observed for genes perturbed in the opposite direction (Fig. 1B).

As control compounds that are not known to have strong anti-depressive effects, alvespimycin displayed limited similarity with statins (average Tau = 23.7), while sirolimus showed an average connectivity score of 92.5. However, the high Tau scores between statins and sirolimus appeared to be driven by genes involved in non-lipid metabolic processes (such as protein metabolism and nucleobase-containing compound metabolism), showing no enrichment of immune processes (Supplementary Fig. 10). This demonstrates the specificity of the pathways commonly perturbed by statins and antidepressants.

### Replication of analysis in NPC cells

Compared to HA1E cells, the same statin compounds induced weaker but predominantly concordant gene expression changes in the NPC cells, with the exception of lovastatin (indicated by a negative Pearson correlation coefficient between its NPC and HA1E gene expression signatures) (Supplementary Fig. 11). The majority of statins showed highly correlated gene expression signatures to each other in NPC cells, with the exceptions of mevastatin, which showed a negative Pearson correlation coefficient to the gene expression signatures of lovastatin and atorvastatin (Supplementary Fig. 12). Similar to HA1E cells, genes perturbed by statins in NPC cells were also functionally related to various biological pathways, including metabolic processes (such as lipid metabolic process) and immune system processes (Supplementary Fig. 13). When more stringent thresholds (|z| > 1.5 or 2) were applied, fewer biological processes were identified to be significantly enriched, and specialized pathways, such as immune system processes and localization, were no longer identified, suggesting an attenuated impact of statins on these specialized pathways in NPC cells (Supplementary Fig. 13).

**Fig. 2:**
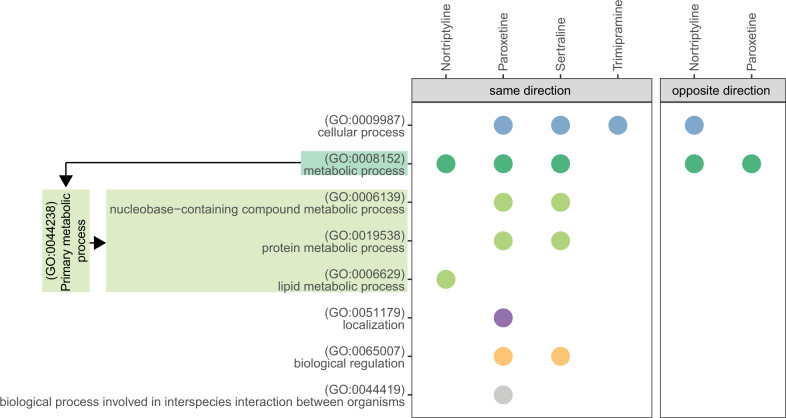
Biological processes identified among genes perturbed by statins and antidepressants in NPC cells. Biological processes identified amongst genes perturbed in the same (left panel) and opposite (right panel) directions by statins and antidepressants are shown. *Y*-axis shows the ancestor GO biological process terms, as well as the child terms of primary metabolic process (GO:0044238) (a child term of “metabolic process”). Arrows indicate ancestor-to-child relationships between GO terms. The bubble plots show the antidepressants for which the biological processes were identified as significantly enriched (gSCS-corrected *p*-value < 0.05) in at least one statin-antidepressant pair.

Similar to HA1E cells, antidepressants were significantly enriched amongst compounds with highly similar gene expression signatures to statins in NPC cells (*p* = 0.0046) (Supplementary Table 6). Although we observed overall weaker gene expression correlation between statins and antidepressants in NPC cells when compared to HA1E cells, several statin compounds, namely fluvastatin and rosuvastatin, induced positively correlated gene expression signatures to all five antidepressants analyzed (Supplementary Fig. 14). Genes perturbed in the same direction by statins and antidepressants in NPC cells were functionally enriched in various biological processes, ranging from cellular and metabolic pathways to localization (Fig. 2). Unlike HA1E cells, we did not observe an enrichment of immune system processes amongst these concordantly perturbed genes (Fig. 2). However, this could be due to the overall more subtle impact of statins on immune system processes in NPC cells, as described above.

### Genetically predicted statin on-target and off-target inhibition is associated with changes in haematological traits

**Fig. 3:**
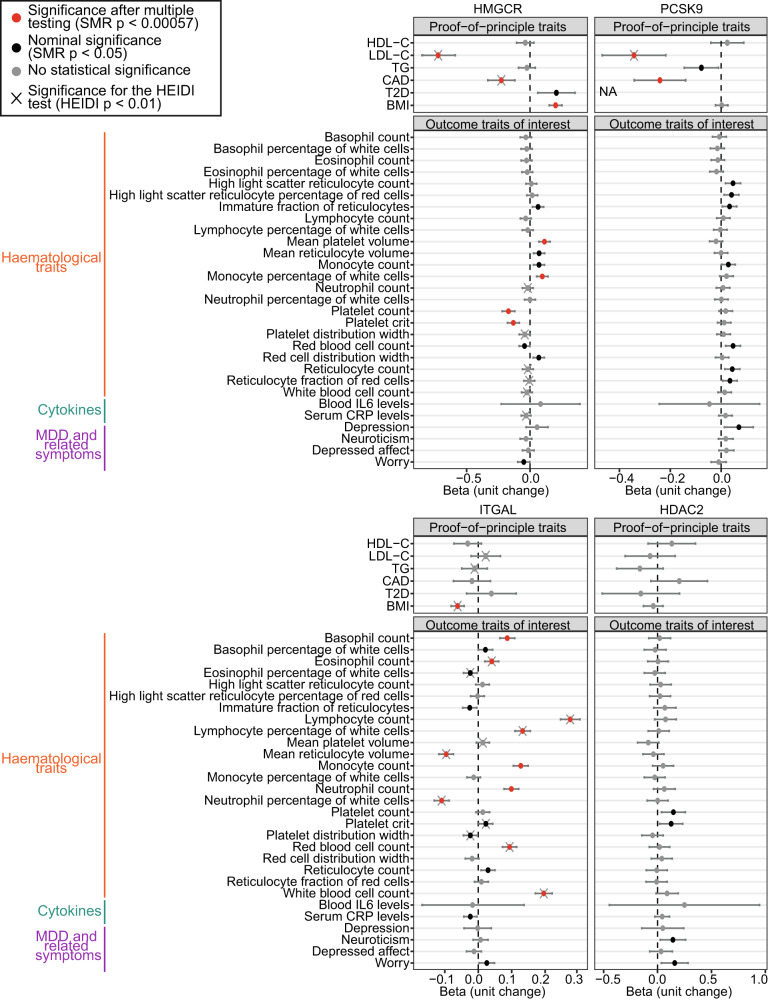
MR analyses of *HMGCR*, *ITGAL, HDAC2* and *PCSK9* gene expression in blood with various haematological, cytokine and depression-related traits. Dot plots show the associations (beta) between gene expression and traits, and error bars show the 95% confidence intervals. The effect sizes are harmonized to represent the changes in trait per one standard deviation decrease in gene expression (thus reflecting genetically proxied target inhibition). The units of beta values are not standardized. Red dots represent associations with statistical significance after multiple testing correction (SMR *p* < 0.00057), and black dots represent associations with nominal significance (SMR *p* < 0.05). Associations with significant HEIDI *p*-values (HEIDI *p* < 0.01) are marked by ×. For *PCSK9*, the genetic instrument was not profiled in the type II diabetes (T2D) dataset and the result was thus labelled with “NA” (“not available”).

Concordant with previous findings, MR analysis showed that reduced *HMGCR* expression in blood was associated with reduced LDL-C [4], reduced CAD risk [41], increased BMI [42] and nominally associated with increased T2D [43] (Fig. 3 and Additional File 6), demonstrating the suitability of this approach for investigating the effects of HMGCR inhibition.

We did not observe an association of genetically predicted HMGCR inhibition with altered risk of depression, neuroticism or depressed affect; however, we found nominal association with reduced worrying symptoms (β = −0.05 (95% confidence interval (CI) = −0.097 to −0.0018); *p* = 0.042) (β values represent change in outcome trait per 1-standard-deviation (SD) decrease in *HMGCR* gene expression) (Fig. 3). MR analysis uncovered significant associations between genetically predicted HMGCR inhibition and changes in haematological traits, including a positive association with mean platelet volume (β = 0.11 (95% CI = 0.069 to 0.16); *p* = 4.49E-07), and a negative association with platelet count (β = −0.17 (95% CI = −0.22 to −0.12); *p* = 1.35E-11) and platelet crit (volume occupied by platelets in the blood as a percentage) (β = −0.13 (95% CI = −0.18 to −0.085); *p* = 3.14E-08) (Fig. 3). In addition, the reduction in *HMGCR* expression showed a significant association with increase in monocyte percentage (β = 0.097 (95% CI = 0.052 to 0.14); *p* = 2.18E-05). We observed no association between *HMGCR* expression and levels of inflammatory cytokines IL6 and CRP. Sensitivity analysis using *HMGCR* expression in brain showed concordant patterns of associations with haematological and depression-related traits (Supplementary Fig. 15 and Additional File 7).

Interestingly, despite extensive validation of the effects of HMGCR inhibition on cardiometabolic traits by previous studies [44, 45], we observed HEIDI significance for the association of *HMGCR* expression in blood with CAD and LDL-C (Fig. 3), which suggested that the variant associated with *HMGCR* expression was distinct from those associated with CAD risk and LDL-C levels. However, this is likely due to complex LD structures in eQTL and GWAS studies (Supplementary Fig. 16), which is a known issue of the HEIDI test.

In analyses of off-target effects, we observed no association between *ITGAL* and *HDAC2* blood expression with lipid traits, confirming that the lipid-lowering effects of statins were driven by on-target HMGCR inhibition (Fig. 3). We found significant associations of genetically proxied ITGAL inhibition with various haematological traits, such as basophil, monocyte and neutrophil counts (Fig. 3 and Additional File 8). Limited associations were found between *HDAC2* expression and outcome traits assessed in this study (Fig. 3 and Additional File 9). Unlike HMGCR inhibition, after multiple testing correction, we observed no significant association with platelet traits for genetically predicted ITGAL or HDAC2 inhibition, suggesting that any potential effect of statins on platelet measures was likely mediated by on-target HMGCR inhibition. Overall, genetically proxied statin target inhibition was found to be associated with changes in diverse haematological traits through both on-target and off-target effects.

To assess whether the observed associations were specific to statins and not other LDL-C-lowering drugs, we performed SMR analysis to predict the effects of Proprotein Convertase Subtilisin/Kexin type 9 (PCSK9) inhibitors (Supplementary Methods, Fig. 3 and Additional File 10). As expected, genetically predicted PCSK9 inhibition showed a significant association with reduced LDL-C and CAD risk, but no significant association with monocyte or platelet traits, indicating that the latter were likely to be independent of lipid-lowering effects of statins. Interestingly, genetically predicted PCSK9 inhibition was nominally associated with increased depression risk, consistent with previous findings [46].

## Discussion

**Fig. 4:**
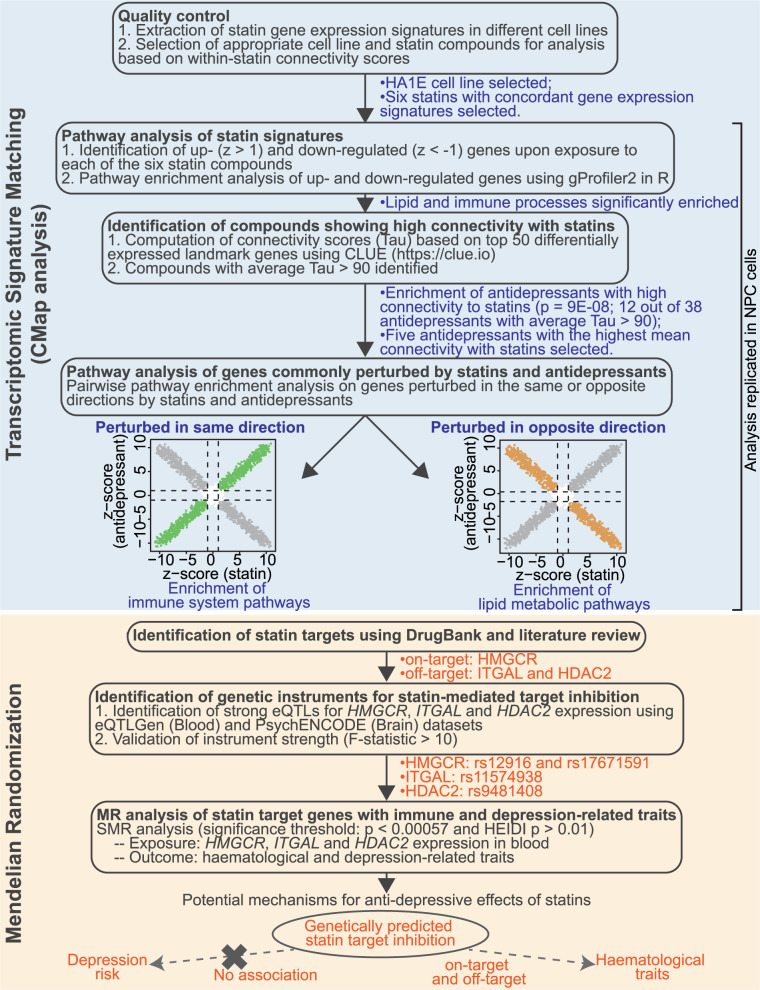
Summary of study design and main findings. We explored the gene expression changes induced by statins using CMap gene expression signatures, and identified biological processes perturbed by statins and antidepressants in the same or opposite direction. We performed Mendelian randomization (MR) analysis to investigate the association of genetically predicted statin target inhibition with various haematological and depression-related traits.

To our best knowledge, this is the first study that directly compares and explores the commonality in the cellular gene expression responses to statin and antidepressant exposure, and provides transcriptomic evidence for shared pharmacological mechanisms of the two drug classes. Using drug-induced gene expression signatures in HA1E cells, we found substantial similarities in gene expression changes induced by statin and antidepressant exposure, conferred by concordant impacts on cellular processes, biological regulation and diverse immune pathways, suggesting a shared pharmacological effect of both drug classes. We observed attenuated effects of statins on immune processes in NPC cells, a model that was biologically more relevant to depression. Using MR, we found no genetic association of HMGCR inhibition with depression risk, but observed extensive associations with various blood cells traits, particularly monocytes and platelets, further supporting an immunomodulatory activity of statins. Our study design and main findings are summarized in Fig. 4. Our findings highlight the importance of future pre-clinical studies to explore the causal role of these biological processes and haematological biomarkers in depression, their clinical value as therapeutic targets in treating depression, as well as their utility in identifying subcohorts of patients with depression (such as those with an inflammatory phenotype) in clinical trials, who are more likely to benefit from immunomodulatory or anti-inflammatory treatments like statins.

Corroborating our results, previous MR studies found no association between LDL-C and depression [47, 48]. Another MR study reported an association between genetically proxied HMGCR inhibition and increased depression risk (using genetic instruments in/near the *HMGCR* gene that were associated with LDL-C levels); however, given they combined the effects from multiple genetic instruments that are in moderate LD (r^2^ = 0.39 in European-ancestry population), their reported effect is likely to be inflated [49].

Immune system dysfunction and elevated pro-inflammatory cytokine levels have been linked to disturbed serotonin metabolism and depressive symptoms [50, 51]. Furthermore, conventional antidepressants exhibit immunomodulatory properties, by reducing the levels of pro-inflammatory cytokines [50]. Although we did not find any association of HMGCR inhibition with IL6 and CRP through MR analysis, we found that pathways related to other pro-inflammatory cytokines, namely interleukin-1 and TNF, were frequently enriched amongst genes downregulated by statins (Additional File 3), corroborating previous findings [14]. Furthermore, MR uncovered extensive associations between genetically predicted HMGCR inhibition and alterations in various blood cell parameters, including monocytes and platelets. Although a previous MR study detected null genetic association between platelet parameters and depression risk [52], depressed individuals have been found to exhibit increased platelet activity [53], with platelet activity reduced after antidepressant treatment [54, 55]. Interestingly, platelets are a major reservoir of serotonin in humans [56], and serotonin’s role in depression is supported by the efficacy of selective serotonin reuptake inhibitors for the treatment of depression [57]. At the same time, increased pro-inflammatory state and activation of monocytes has been observed in individuals with depression compared to healthy controls [58].

Despite strong evidence of a role of the immune response and inflammatory biomarkers in depression, previous clinical trials of treatments for depression that specifically target these pathways have largely been unsuccessful [59]. This may be due to an underrepresentation of inflammatory phenotypes within case participants, as individuals with increased inflammation are estimated to make up less than one-third of the depressive population [59]. Though previous RCTs have reported clinical benefits of statins when used as an add-on treatment to antidepressants, limited evidence has supported the beneficial effects of statin monotherapy on treating depression [6]. Stratification of RCT participants based on their immune and inflammatory profiles may potentially be valuable to identify subtypes of depression that are more likely to be responsive to statin treatments. Furthermore, common assessment measures of depression, such as the 17-item Hamilton Depression Rating Scale, incorporate physical, cognitive, and emotional symptoms [60]. Such combined measures may be limited in detecting symptom-specific effects of immunomodulatory drugs. For example, Infliximab, a TNF antagonist, has shown preferential effects on anhedonic symptoms (inability to feel pleasure) [61]. Corroborating these findings, our MR analysis found that genetically predicted HMGCR inhibition was nominally associated with reduced worrying symptoms, while showing no association with the other symptoms assessed. Therefore, better study designs, including cohort stratification based on immune profiles, assessment of relevant inflammatory biomarkers as secondary measures, and assessment of specific symptoms, are required to evaluate the efficacy of drugs with immunomodulatory and anti-inflammatory properties, such as statins, on treating depression.

Some statins exhibit in vitro off-target inhibition of ITGAL and HDAC2 [29–32], although these may not necessarily translate to in vivo pharmacological actions. While we observed no association with depression or depressed affect, genetically predicted ITGAL and HDAC2 inhibition was nominally associated with increased worrying, which was in the opposite direction to HMGCR inhibition, and increased neuroticism. Although *ITGAL* expression is absent in HA1E cells (based on the DepMap expression data [62]), and therefore unlikely to contribute to the immunomodulatory effects of statins observed in CMap analysis, MR demonstrated that lowering of *ITGAL* expression in blood was associated with extensive immune cell changes, such as basophil, monocyte and neutrophil counts, but was not associated with platelet measures, indicating that the potential effects of statins on platelets were likely mediated by HMGCR inhibition. Furthermore, genetically proxied ITGAL inhibition, but not HMGCR inhibition, was nominally associated with reduced CRP levels, suggesting that the previously reported CRP-lowering effects of statins could potentially be mediated through off-target pathways. Given its expression and functional involvement in diverse immune cell populations [63], inhibition of ITGAL likely leads to extensive alterations in immune phenomena. Taken together, our findings suggest that statins exhibit modulatory effects on the immune system and possibly depressive symptoms through both on-target HMGCR inhibition, and potentially off-target ITGAL inhibition. Given not all statin compounds have evidence for off-target inhibition of ITGAL, further pre-clinical investigation is required to differentiate whether shared pharmacological effects of antidepressants and statins are mediated through on or off-target effects of statins, which will help inform which statin compounds should be investigated for repurposing in the future.

Most previous studies on the association between statins and depression have been observational studies or RCTs [7–12]. Our study offers a genomics approach to investigating these effects at the molecular level, via transcriptomic signature matching and genetic association analysis. We performed MR analysis using large eQTL and GWAS datasets, which is less prone to unmeasured confounder bias and reverse causality than observational studies [64].

Several limitations need to be acknowledged. Firstly, this study is based on in vitro changes in response to drug exposure, which may not accurately reflect in vivo effects, as the human cell lines potentially exhibit genomic variations from biological tissues. In addition, the dosage and length of statin exposure used in CMap experiments might not reflect the doses used in clinical treatment or the ability of the specific statin compound to reach the most relevant tissue for exerting any potential anti-depressive effects. However, gene expression signature matching has been shown to be a cost-effective and feasible approach to uncovering unknown effects of compounds. For example, this approach has been used to identify the autophagy-enhancing effect of Fasudil, a Rho-kinase inhibitor, supporting its repurposing potential as a treatment for neurodegenerative diseases [65]. Fasudil is currently in Phase II clinical trials as a potential treatment for amyotrophic lateral sclerosis [66]. Secondly, while loss-of-function genetic variants or protein quantitative trait loci (pQTL) provide better genetic instruments for statin-mediated target protein inhibition, we were limited by the availability of such genetic variants, and thus used strong and previously validated eQTLs as instruments. However, we have demonstrated known beneficial and adverse cardiometabolic effects of statins using this approach, suggesting that other observed effects from our analysis would be worth further investigation. Additionally, the associations observed for genetic variants provide an estimated effect of lifelong statin exposure and may not reflect short-term effects. However, investigating the long-term effect of statins is clinically relevant, as they are often lifelong medications [67]. Finally, different statin compounds, varying in pharmacokinetic characteristics, display different ability to cross the blood-brain barrier and association with depression [6]. A comparative analysis between different statins may provide insights into the chemical properties underlying their anti-depressive effects.

In conclusion, we show that statins evoke an antidepressant-like effect on various immune activation and response processes in human cell lines, and demonstrate potential modulatory impacts of statins on haematological traits (including those that have previously been linked to depression) through both on-target and potential off-target effects. At the time of writing, there are ongoing clinical trials investigating the effect of statin treatments on depression and mood disorder [68, 69]. Our findings warrant further pre-clinical investigation into whether shared pathways perturbed by both statins and antidepressants play a role in modulating depressive symptoms. Our findings also highlight the necessity for future clinical trials investigating the effects of statins on depressive symptoms, especially in the context of an inflammatory phenotype. Furthermore, the above identified blood biomarkers could be used as secondary outcomes in these clinical trials, and to stratify participants and identify subcohorts that are most likely to respond to statin treatments. Lastly, given not all statins have the same off-target effects, the choice of statin compound may be another important consideration in future pre-clinical and clinical studies.

## Supplementary information

Additional File 1 (.pdf).

Additional File 2 (.xlsx).

Additional File 3 (.xlsx).

Additional File 4 (.xlsx).

Additional File 5 (.xlsx).

Additional File 6 (.xlsx).

Additional File 7 (.xlsx).

Additional File 8 (.xlsx).

Additional File 9 (.xlsx).

Additional File 10 (.xlsx).

## Data Availability

All data generated or analyzed during this study are included in this published article and its additional files. The publicly available CMap dataset was retrieved from the GEO repository (GSE92742). All GWAS summary statistics analyzed in this study were retrieved from original publications or public platforms, as referenced in the manuscript.

## Code availability

Analysis scripts can be found at https://github.com/CNSGenomics/statin_depression.
