# Supplementary material for: Investigating the potential anti-depressive mechanisms of statins: a transcriptomic and Mendelian randomization analysis
Source: Transl Psychiatry. 2023 Apr 4;13:110. doi: 10.1038/s41398-023-02403-8 (PMC10073189; doi:10.1038/s41398-023-02403-8)
Supplement: Supplementary file 1 — Additional File 1 (.pdf). [file 41398_2023_2403_MOESM1_ESM.pdf]

Supplementary Materials

of

Investigating the potential anti-depressive mechanisms of statins: a transcriptomic and Mendelian randomization analysis

Jiayue-Clara Jiang[1] (PhD), Chenwen Hu[2] (Msc), Andrew M McIntosh[3] (MD), Sonia Shah[1] (PhD)

[1] Institute for Molecular Bioscience, The University of Queensland, St Lucia, Australia

[2] The University of Queensland, St Lucia, Australia

[3] Division of Psychiatry, University of Edinburgh, Edinburgh, United Kingdom

Supplementary Methods

CMap gene expression signatures

The Connectivity Map (CMap) database was first introduced in 2006 [1]. The Library of Integrated Cellular Signatures (LINCS) database from 2017 represents a 1 000-fold scale-up of the original CMap database and contains a diverse collection of transcriptomic responses to chemical (e.g. pharmacological compounds) and genetic (e.g. gene knock-down or overexpression) perturbations, primarily profiled using human cell lines [1, 2]. For each perturbagen, its transcriptomic impacts of different treatment dosages and timepoints are investigated using the L1000 assay, which directly measures the gene expression of 978 genes (termed "landmark" genes) upon exposure to perturbagen [2]. The expression of additional 11 350 genes are computationally inferred from the landmark genes, constructing a transcriptomic profile consisting of a total of 12 328 genes [2]. The L1000 assay generates gene expression profiles that show high correlation with those measured using RNA-seq, validating its use as a feasible and cost-effective approach for high-throughput profiling of perturbagen-induced gene expression changes [2].

Selection of statin gene expression signatures

In this study, the level-5 z-scores of the CMap signatures, which represent a normalized measure of the magnitude and direction of gene expression changes induced by chemical or genetic perturbations, were acquired from the GEO repository (GSE92742) in the GCTX format (accessed on July 8th, 2021). The gene expression signatures of statins from the HA1E kidney cell line were selected for detailed analysis, due to the greatest concordance and specificity in gene expression changes amongst different statins (Supplementary Figure 2). Previous validation of the CMap pipeline shows that drugs are able to elicit intended pharmacological responses even in cell lines

that are not derived from their primary target tissue types, provided that their targets are expressed in the cell lines [1]. We queried the Protein Atlas [3] and DepMap expression platforms [4], and confirmed that HMGCR, the primary target of statins, was ubiquitously expressed in diverse human tissues and cell lines, including kidney and brain. Detailed analysis of statin gene expression signatures was performed on HA1E signatures generated in selected experimental conditions (time = 24 h, dose = 10 μM). The gene expression signatures of antidepressants in HA1E cells were also retrieved to enable analysis of the differentially expressed genes shared by both statins and antidepressants. The gene expression signatures of alvespimycin (heat shock protein inhibitor) [5] and sirolimus (mTOR inhibitor) [6] were analyzed as controls, as they were reported by the CMap team to induce consistent and strong transcriptomic responses across cell lines, and are not functionally linked to strong anti-depressive effects. Limited evidence suggests a cholesterol-modifying effect of sirolimus [7, 8], with little evidence on the effect of alvespimycin on lipids.

CMap NPC signatures

NPC cells are undifferentiated progenitor cells that give rise to glial and neuronal cell types in the central nervous system [9]. In this study, as a sensitivity analysis, we analyzed the CMap gene expression signatures of statins in NPC cells as they are biologically more relevant to depression. Previous analysis of the CMap data reported that out of a total of 189 compounds that induced consistent gene expression changes (indicated by high connectivity scores) in neuronal cell lines, 34% showed similar gene expression signatures between the NPC cells and the nine core cancer cell lines (details on core cell lines presented below), compared to 50% that showed similar gene expression signatures amongst the nine cancer cell lines [2]. This indicates that while for some compounds, their gene expression signatures from the nine cancer cells can be used as proxy for

their signatures in brain cell lines, a large proportion of compounds may induce different gene expression changes in neuronal cell lines [2]. However, while these differences may be due to biological variations between neuronal and cancer cells, it remains unclear how well NPC cells proxy for drug response in neurons. It is also important to note that NPC cells are less well profiled compared to the nine core cancer cell lines, and unlike the generally homogenous cancer cell lines, NPC cells are highly heterogenous cell populations with greater diversity in molecular and transcriptomic characteristic [9].

CMap connectivity scores

In this study, to compute the connectivity profile of statins using the CMap platform, we constructed the query signatures of statins using the top *n* most up-regulated and top *n* most down-regulated landmark genes selected from their CMap gene expression signatures (Supplementary Figure 1). While the selection of *n* is arbitrary, we used *n* = 50 for the primary analysis, as the pre-compiled Tau scores between reference signatures in the CMap database, which are available on the online CLUE platform (https://clue.io), are computed using the top 50 up-regulated landmark genes and the top 50 down-regulated landmark genes. We used *n* of 100 and 150 in sensitivity analyses.

The CMap platform uses an algorithm based on the weighted Kolmogorov-Smirnov enrichment statistic to quantify the similarity between a query signature and a reference CMap signature where the genes are ranked from the most up-regulated (high ranking) to the most down-regulated (low ranking) [2] (Supplementary Figure 1). The degree of connectivity is computed as Tau scores, which represent a statistical measure of the likelihood of observing the similarity between the query and reference signatures, given all transcriptomic signatures in the reference database. The

Tau scores range between -100 and 100. A positive Tau score indicates positive connectivity, meaning that genes up-regulated in the query signature predominantly have high ranking in the reference signature, and genes down-regulated in the query signature predominantly have low ranking in the reference signature (Supplementary Figure 1). In contrast, a negative Tau score indicates negative connectivity, meaning that the query gene expression signature is essentially 'reversed' in the reference signature, i.e. up-regulated genes in the query signature predominantly have low ranking in the reference signature, and down-regulated genes in the query signature predominantly have high ranking in the reference signature (Supplementary Figure 1). For example, one would expect an agonist and an antagonist for the same target to have high negative Tau scores.

A subset of the CMap dataset, termed the "Touchstone" collection, contain gene expression signatures of over 8,000 well-annotated chemical and genetic perturbagens, profiled in a "core" set consisting of nine cancer cell lines (A375, A549, HA1E, HCC515, HEPG2, HT29, MCF7, PC3, and VCAP). The Touchstone reference dataset is used in the CLUE platform as the reference set against which queries are compared to compute connectivity profiles. For each query signature, the CLUE algorithm computes a connectivity profile for each of the nine core cancer cell lines (Supplementary Figure 1). For example, a connectivity profile from HA1E cells indicate the connectivity between the query signature and the reference gene expression signatures generated by exposing HA1E cells to the perturbagens. CLUE also computes a summary connectivity profile, as described by Subramanian et al. [2], which indicates the connectivity between the query signature and the reference signatures summarized across cell-type-specific connectivity scores (Supplementary Figure 1).

Transcriptome-wide pairwise correlation

We also interrogated the correlation between a pair of signatures for all 12 328 genes, using Pearson correlation in R.

Annotation of biological process terms with ancestor terms

Gene Ontology (GO) biological process terms are placed in a hierarchical structure consisting of parent/child term relationships [10]. While functional annotation by gProfiler2 provides detailed information on the enriched biological processes, the resulting list of significant terms often contains overlapping and highly redundant biological processes, confounding the interpretation of results. We thus summarized the GO biological process terms based on their high-level ancestor terms. Using GO.db (version 3.13.0) [11], we retrieved the child terms of biological process (GO:0008150), which is the root term of all GO biological pathway terms, and defined these child terms as the list of ancestor biological process categories used for downstream annotation. We annotated each statistically significant GO biological process term based on their ancestor categories. Each GO term might be annotated with more than one ancestor category. Similarly, we also categorized the GO biological terms into primary metabolic process (GO:0044238) (a child term of "Metabolic process") and immune system process (GO:0002376) terms.

Annotating antidepressants

The Anatomical Therapeutic Chemical (ATC) classification system provides a reference for drugs, which are hierarchically categorized based on their targeted organs as well as their therapeutic, pharmacological and chemical attributes [12]. While we acknowledge that there are non-ATC-profiled drugs or active substances with anti-depressive properties, in this study we limited our analysis to antidepressants documented by the ATC system (ATC code: N06A) as they were more likely to have established efficacy and thereby provided a valid basis for interrogating the

molecular mechanisms underlying their anti-depressive effects. A total of 38 ATC-documented antidepressants are profiled by the CMap database (Supplementary Table 3).

Identifying shared perturbed pathways between statins and antidepressants

We hypothesized that if statins and antidepressants indeed exhibited shared pharmacological effects, they would likely perturb the same biological pathways. Out of the 38 antidepressants profiled in CMap, we selected five antidepressants (desipramine, nortriptyline, paroxetine, sertraline and trimipramine) for further analysis as they demonstrated the highest average connectivity with the six statins studied in HA1E cells. We performed pairwise comparison of the transcriptomic signatures of six statins against the five antidepressants, which gave rise to a total of 30 statin-antidepressant combinations. For each pair of statin and antidepressant, genes that were differentially expressed (defined by $|z| > 1$) in both signatures were further categorized into genes perturbed in the same or opposite direction. Using gProfiler2 and parameters described in Methods, pathway enrichment analysis was performed for each category. Similarly, we also performed pairwise pathway enrichment analysis for genes commonly perturbed by statins and the two control drugs (alvespimycin and sirolimus).

Mendelian randomization

The principle underlying Mendelian randomization (MR) originates from Mendel's laws of segregation and independent assortment. By Mendel's laws, alleles randomly segregate and are passed from parents to offspring during the process of meiosis, and the inheritance of alleles of one variant occurs independently of alleles of other variants [13]. In randomized controlled trials, participants are randomly allocated to the treatment or control groups, and thereby subjected to different levels of exposure. MR uses genetic instruments linked to the exposure of interest as

proxies, as if the level of exposure is randomly allocated at conception. This random assignment of genetically determined exposure at conception is less likely to be affected by various lifestyle and socioeconomic factors, which may otherwise distort the exposure-outcome associations [14, 15]. Furthermore, as the genetic determinants for exposures are generally fixed at conception and thus not affected by the outcome, MR is less prone to reverse causation, which may be problematic to dissect using observational studies [16]. Genetic variants are only valid instrument variables for MR analysis if they meet three key assumptions, that they are strongly associated with the exposure, not associated with any confounder of the exposure-outcome association, and are associated with the outcome only through the exposure of interest (no horizontal pleiotropy) [17].

The selection of genetic instruments depends on the genetic architecture of the exposure [18]. For polygenic traits, such as low-density lipoprotein cholesterol (LDL-C) levels, the genetic proxy for these traits is derived from genetic variants from multiple trait-associated loci [18]. In contrast, in cases where the exposure is a protein, such as HMGCR, its genetic instruments may be selected from loss-of-function mutations, expression quantitative trait loci (eQTL) or protein quantitative trait loci (pQTL) located within or nearby the protein-encoding gene [18]. Previous studies have reported an enrichment of eQTLs in single nucleotide polymorphisms (SNP) associated with complex traits, and suggested gene expression as an important mediator of SNP-trait associations, supporting the utility of eQTLs in identifying genetic determinants of traits and diseases [19, 20]. Furthermore, eQTLs are widely used as genetic proxies for drug targets in MR analyses [21, 22].

In this study, we assessed the strength of genetic instruments using the F-statistics from the linear regression model of the genetic instruments with the exposure (gene expression). We determined SNPs with F-statistic > 10 to be suitable genetic instruments, where F-statistic was calculated as previously described [18, 21].

eQTLGen blood eQTLs

The eQTLGen consortium has produced an extensive catalogue of both *cis* and *trans* eQTLs for human gene expression in blood [23]. The blood eQTL data were generated via meta-analysis of over 31 684 samples from 37 cohorts consisting of primarily European-ancestry individuals [23]. In the current study, only cis-eQTLs were considered for the selection of genetic instruments.

PsychENCODE eQTLs

To use genetic instruments relevant to the biology of depression, we retrieved brain eQTL data produced by the PsychENCODE consortium. The PsychENCODE eQTL dataset contains prefrontal cortex eQTLs identified in a cohort of 1 387 individuals [24].

Genetic instrument for HMGCR inhibition

To investigate the on-target effect of statins, we selected rs12916 as the genetic proxy for HMGCR inhibition. The rs12916 SNP is located in the 3′ untranslated region of the *HMGCR* gene, and the genetic associations between the rs12916-T allele and metabolomic traits were shown to mirror the actual effects of statin exposure on the metabolome [25]. It is widely used as a genetic proxy for assessing statin effects [26, 27, 28]. Rs12916 is a strong eQTL for *HMGCR* expression, where each additional rs12916-T allele is associated with 0.1 standard deviation (SD) decrease in *HMGCR* expression in blood (eQTLGen: p = 1.5E-36). As a sensitivity analysis, we also selected rs17671591, a strong eQTL (p = 2.5E-05) for *HMGCR* expression in the brain prefrontal cortex (PsychENCODE [24]), as the genetic instrument for HMGCR inhibition in the brain. Rs17671591 is in moderate linkage disequilibrium (LD) ($r^2 = 0.6$) with rs12916 amongst European-ancestry individuals.

Statin off-target inhibition of ITGAL and HDAC2

We used the DrugBank database [29] to identify off-target effects of statins, and found that selective statins exhibited *in vitro* inhibition of Integrin Alpha-L (ITGAL) and Histone Deacetylase 2 (HDAC2). Although atorvastatin has been reported to exhibit additional off-target effects (namely DPP4, AHR and NR1I3) [29], these off-target effects are unique to atorvastatin, and thus were not investigated in this study.

ITGAL is a component of the heterodimeric Lymphocyte Function-associated Antigen-1 (LFA-1) receptor, which is expressed in diverse immune cell populations and plays a role in modulating cell adhesion [30, 31]. Amongst the statins investigated in this study, lovastatin, rosuvastatin and simvastatin are found to show *in vitro* binding to ITGAL, subsequently inhibiting LFA-1 function and T-cell adhesion, proliferation, and cytokine production [32, 33, 34]. At the same time, a subset of statins (atorvastatin, fluvastatin, lovastatin, pravastatin and simvastatin) are also found to exhibit *in vitro* inhibition of HDAC2 [35], which is involved in chromatin remodeling [36].

Using the eQTLGen data [23], we selected the most significant eQTLs in blood to proxy for inhibition of ITGAL and HDAC2. Specifically, each additional rs11574938-C allele was associated with 0.21 SD decrease of *ITGAL* expression (p = 7.9E-150), and each rs9481408-T allele was associated with 0.048 SD decrease of *HDAC2* expression (p = 4.1E-07) in blood.

Genetic instrument for PCSK9 inhibition

We sought to investigate whether any genetic associations observed for statin targets were likely to be mediated independently of cholesterol lowering, by extending MR analysis to other lipid-lowering medications, namely Proprotein Convertase Subtilisin/Kexin type 9 (PCSK9) inhibitors

[37]. *PCSK9* eQTLs were absent in the eQTLGen dataset, as *PCSK9* was likely removed from meta-analysis due to the lack of variation in expression. We thus identified *PCSK9* eQTLs from the GTEx eQTL dataset. The GTEx project (version 8) identified and profiled eQTLs from diverse tissue types [38]. The majority of the GTEx cohort consists of individuals of European ancestry. We retrieved *PCSK9* eQTLs identified in whole blood, which contains 670 samples, and performed MR analysis using a strong eQTL (rs12117661; $p = 8.3E-11$) as the genetic instrument for *PCSK9* expression. This eQTL was previously found to be moderately associated with plasma PCSK9 protein expression ($p = 0.00029$) [39].

HEIDI test

To validate that the exposure-outcome association was mediated through one single causal SNP, rather than via LD between separate SNPs (linkage scenario), the Heterogeneity in Dependent Instruments (HEIDI) test was performed as a sensitivity analysis [40]. Without correcting for multiple testing, we used a HEIDI p-value threshold of 0.01 to define statistical significance, which was a stringent threshold. A HEIDI p-value > 0.01 indicated that the estimated effect of exposure on outcome was likely due to single causal variants, while a p-value < 0.01 suggested that the observed association was potentially attributable to the linkage scenario.

LocusCompare plots

LocusCompare plots allow the visualization of the distribution of genome-wide association studies (GWAS) or eQTL summary statistics [41]. Using the LocusCompareR package (version 1.0.0) [41], we generated the LocusZoom and significance ($-\log_{10}P$) scatter plots for LDL-C and *HMGCR* eQTLs from eQTLGen. The LD scores were generated based on the European-ancestry population.

Supplementary Figures

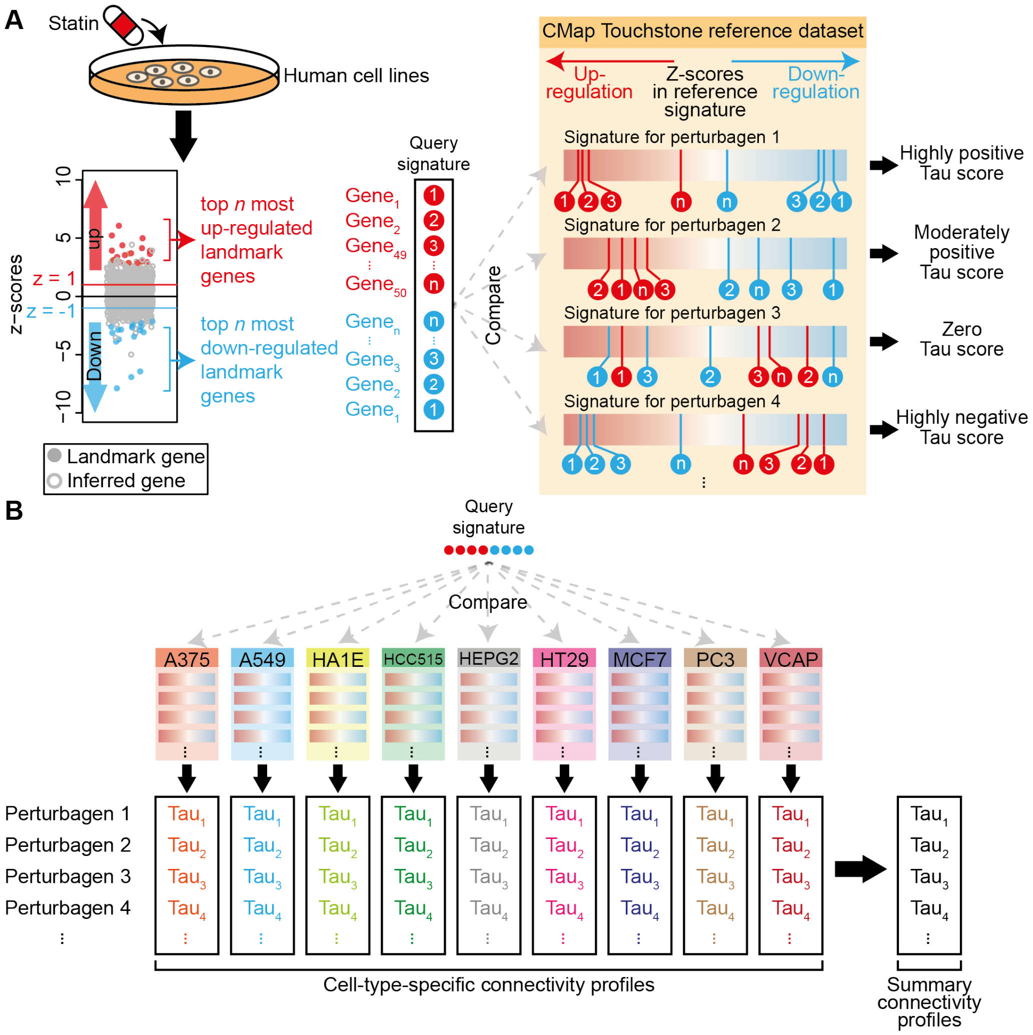

Supplementary Figure 1. Schematic illustration of statin connectivity profile generation. (**A**) For each perturbagen of interest (e.g. a chemical compound), its gene expression signature is generated by exposing human cell lines to the perturbagen. In this study, the gene expression signatures of statins were retrieved from the CMap dataset (GSE92742). To compute the connectivity profile of each statin signature, a query signature is contructed consisting of n most up-regulated (z-score > 1) landmark genes (referring to genes directly measured in the L1000 array) and n most down-regulated (z-score < -1) landmark genes from the statin gene expression signatures. Using the online CLUE algorithm, the query signature is compared against every perturbagen gene expression signature from the Touchstone reference dataset. Reference signatures that show concordant gene expression changes to the query signature are assigned a positive connectivity (Tau) score, while reference signtures that show discordant gene expression changes to the query signature are assigned a negative connectivity score. (**B**) For each query signature, the CLUE algorithm computes cell-type-specific connecivity profiles, generated by comparing the query signature against the reference signatures profiled in each cell line seperately. By summarizing the connectivity profile across the nine core cell lines, CLUE also computes a summary connecivity profile.

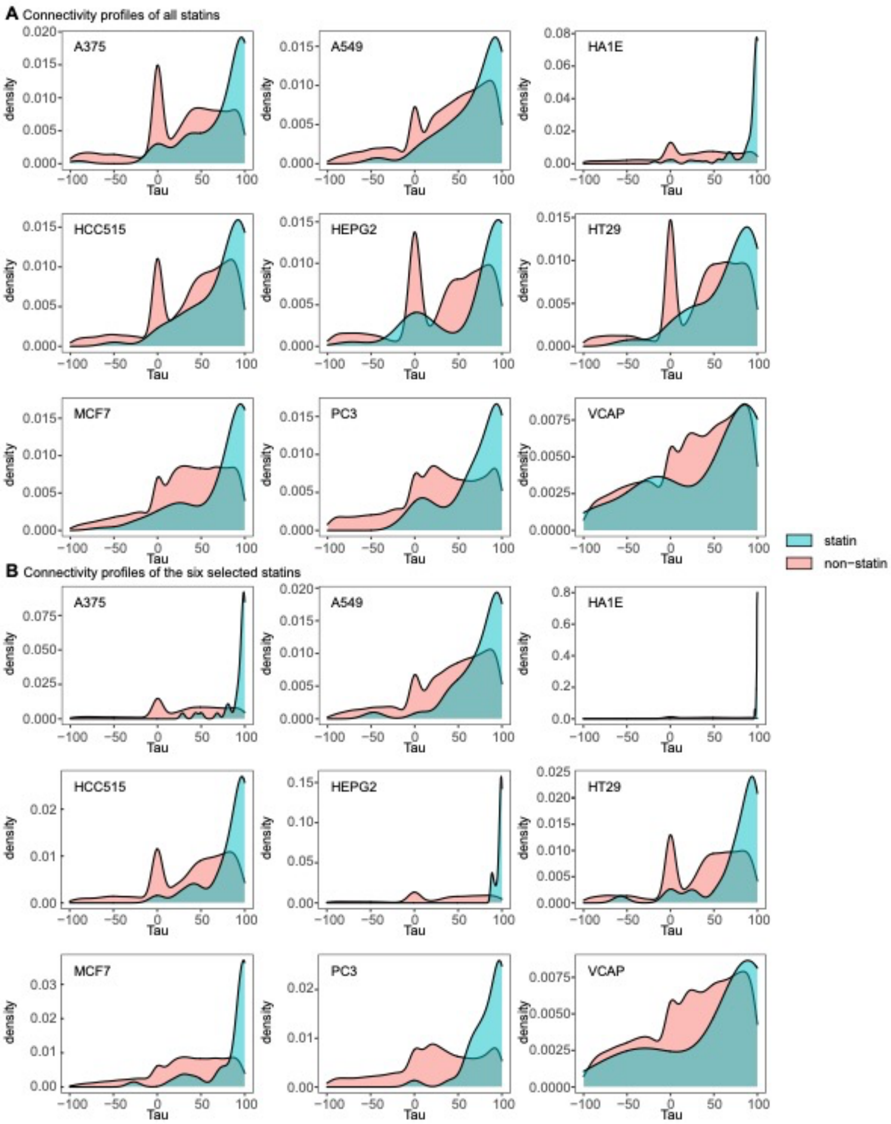

Supplementary Figure 2. Kernel density plots of connectivity (Tau) scores computed between different statin compounds (blue), as well as between statins and non-statin compounds (pink) in various cell lines. (**A**) Tau score distribution for all statins (a total of 9, not measured in every cell line) profiled in the CMap database. Compared to the other cell lines, statin-induced gene expression signatures displayed the most concordance in HA1E cells reflected by overall high between-statin connectivity scores (blue), and high specificity, reflected by low connectivity with most other compounds (pink). (**B**) Tau score distribution for six statins (atorvastatin, fluvastatin, lovastatin, mevastatin, rosuvastatin and simvastatin) included in detailed analysis, which were chosen as they were profiled in HA1E cells under selected exposure conditions (time = 24 h and dose = 10 μM). Pravastatin was not included in analysis as it showed poor connectivity with other statin compounds. The connectivity scores shown in these plots were obtained from the Touchstone dataset, pre-compiled by CLUE (https://clue.io) [2].

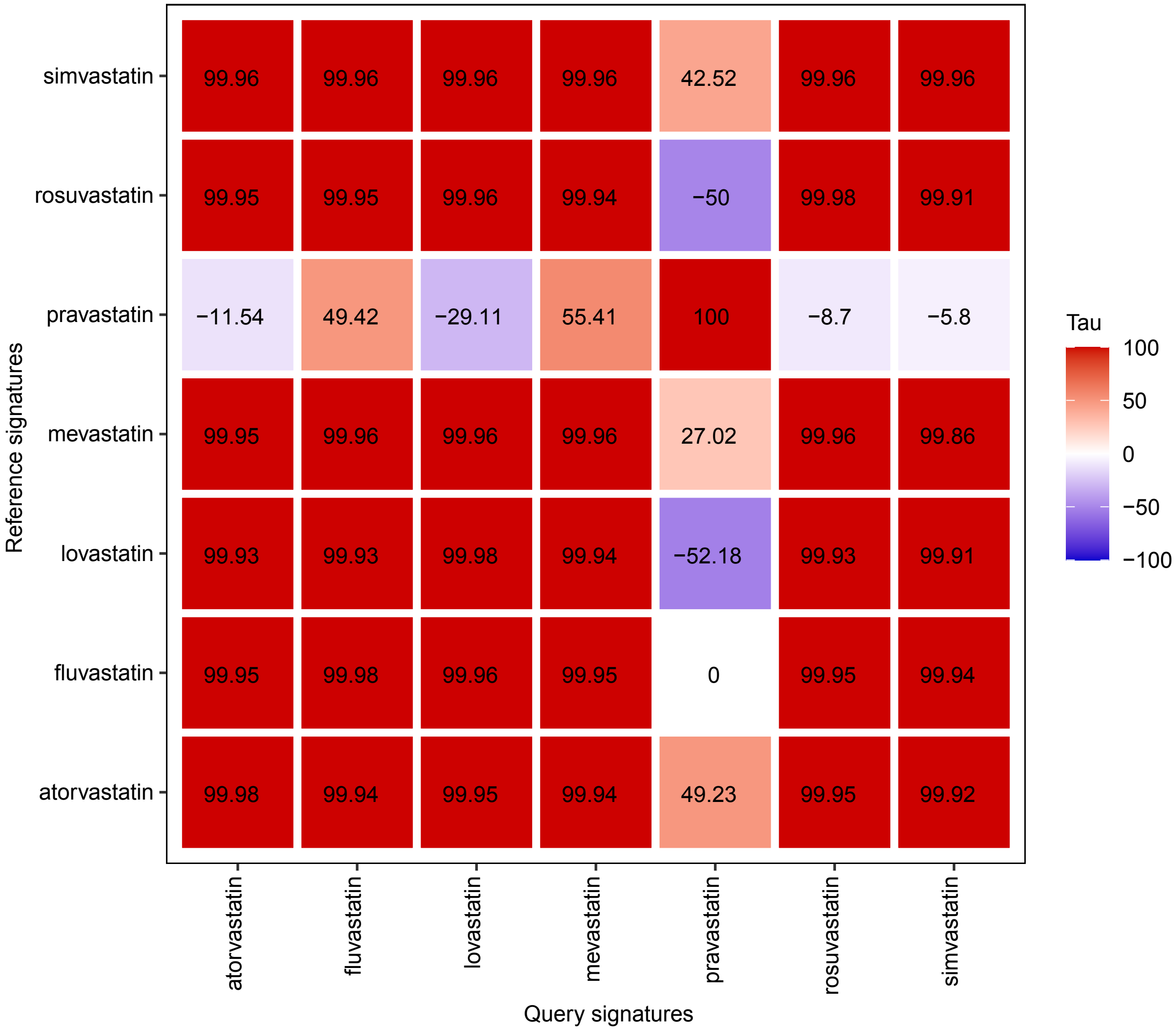

Supplementary Figure 3. Connectivity scores amongst statin signatures in HA1E cells. The connectivity scores shown here were generated by submitting statin HA1E query signatures to CLUE (https://clue.io). Columns show the statin query signatures, and rows show the reference statin signatures in the CMap database. The corresponding connectivity scores (Tau scores) are annotated on the heatmap.

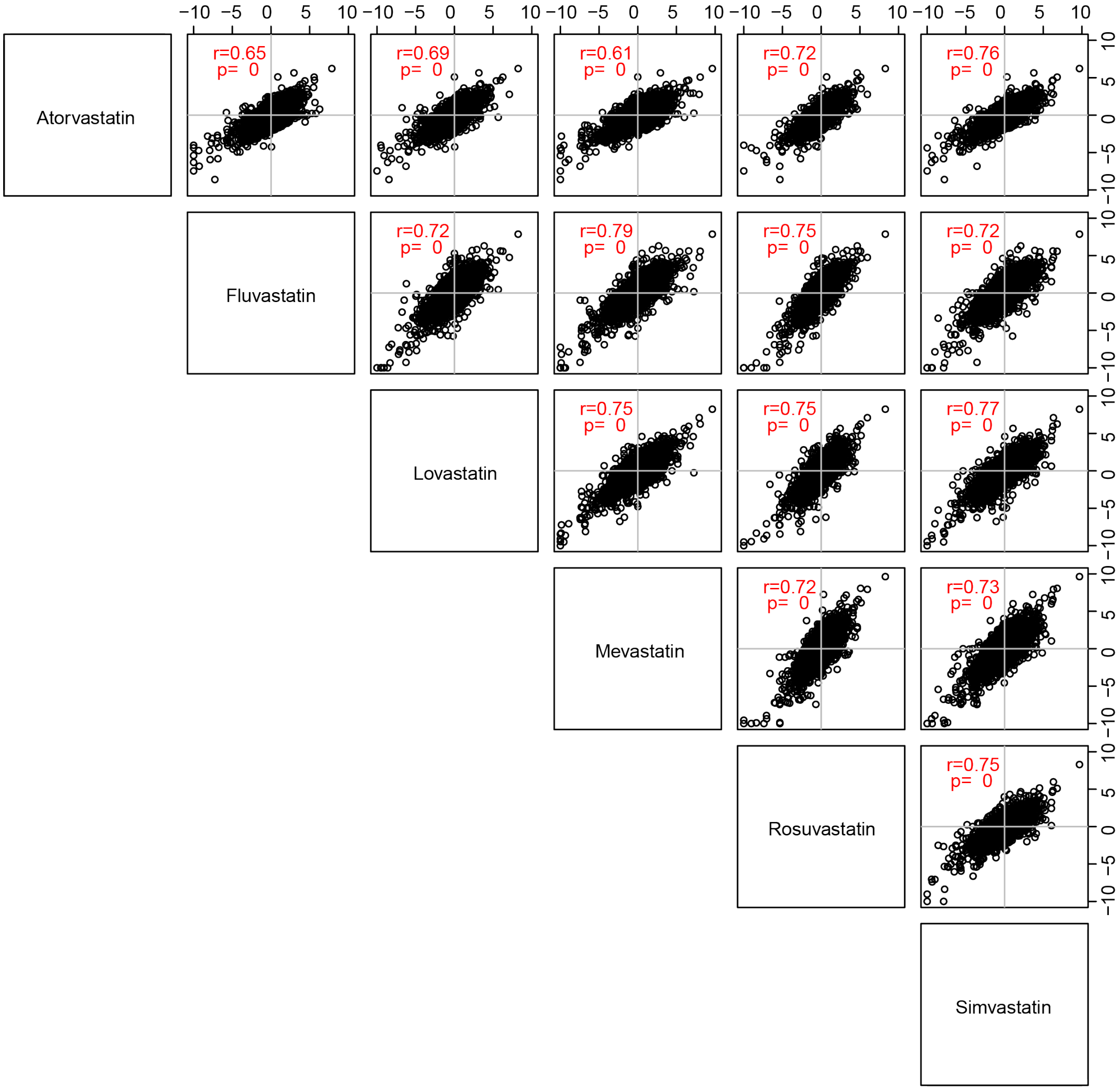

Supplementary Figure 4. Statins induced concordant transcriptional responses in HA1E cells. Changes in gene expression (z-scores) of 12 328 genes profiled by CMap were compared for each pair of statins using pairwise Pearson correlation. The Pearson correlation coefficients (r) and the corresponding two-sided p-values (p) are shown.

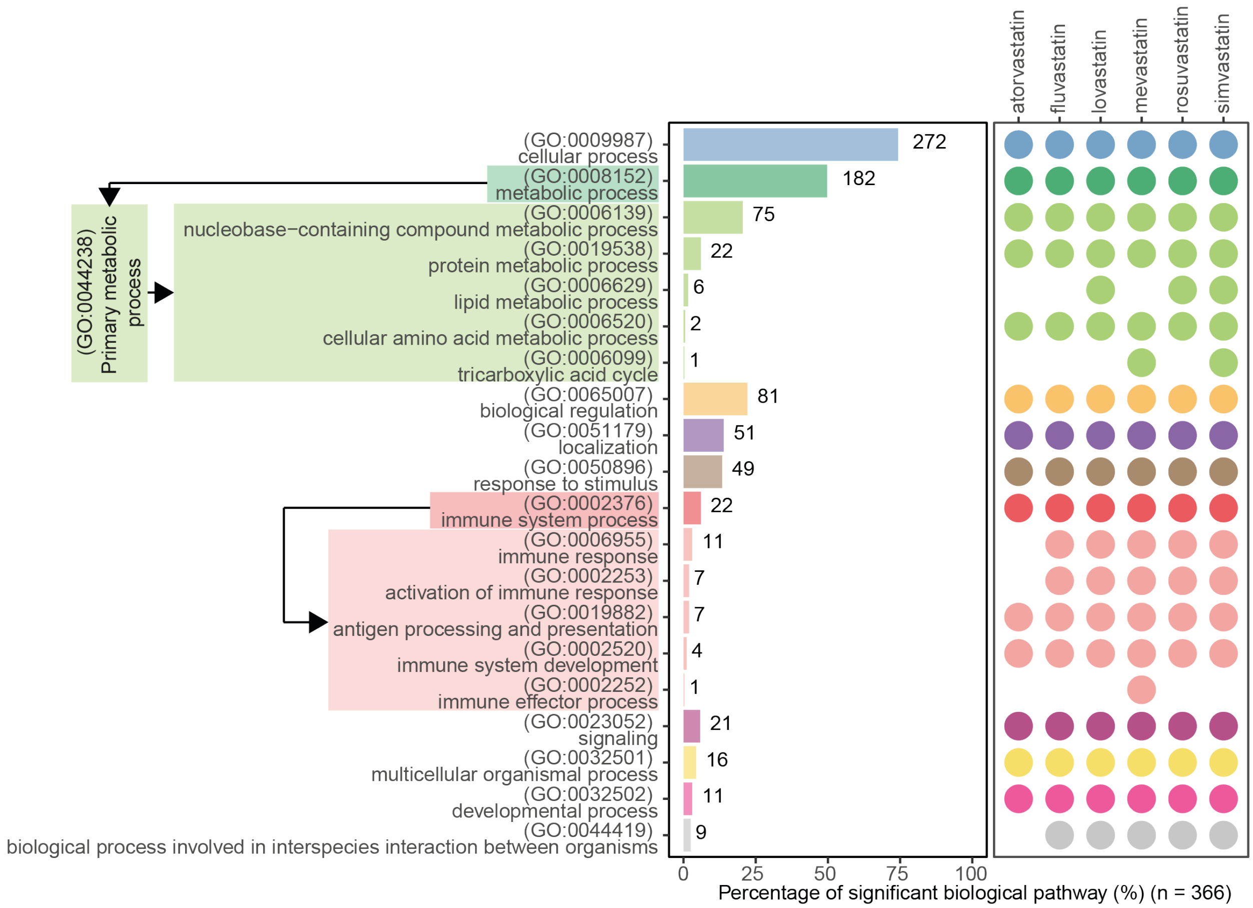

Supplementary Figure 5. Genes functionally involved in lipid and immune pathways were widely perturbed by statin exposure in HA1E cells. A total of 366 biological process terms were identified as significantly enriched amongst genes up-regulated or down-regulated ($|z| > 1$) by at least one statin, and categorized into high-level ancestor terms. Y-axis shows the ancestor biological process terms, as well as the child terms of primary metabolic process (GO:0044238) (a child term of "metabolic process") and immune system process (GO:0002376). The arrows indicate ancestor-to-child relationships of GO terms. Bar graph shows the percentages of significant GO biological process terms annotated with each ancestor terms, with the corresponding counts shown on the graph. The bubble plot shows the statin compounds for which the biological processes were identified as significantly enriched.

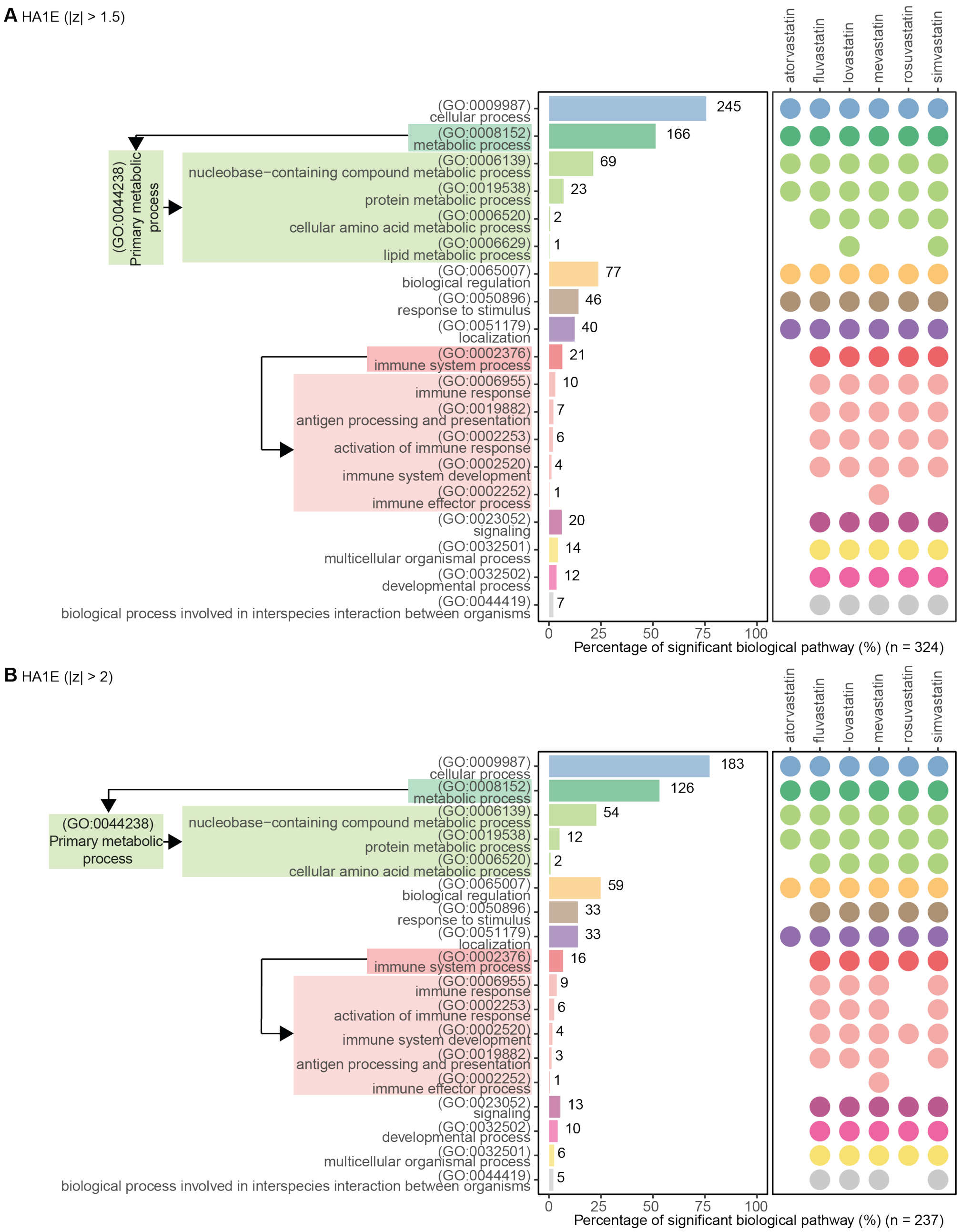

Supplementary Figure 6. Pathway enrichment results using more stringent z-score thresholds for defining differentially expressed genes. Pathway enrichment analysis was performed for differentially expressed genes, defined by (**A**) absolute z-score > 1.5 and (**B**) absolute z-score > 2, where a total of 324 and 237 biological pathways were identified as significantly enriched respectively. Enriched biological process terms were categorized into high-level ancestor terms. Y-axis shows the ancestor biological process terms, as well as the child terms of primary metabolic process (a child term of "metabolic process") and immune system process. The arrows indicate ancestor-to-child relationships of GO terms. Bar graph shows the percentages of significant GO biological process terms annotated with each ancestor term, with the corresponding counts shown on the graph. The bubble plot shows the statin compounds for which the biological processes were identified as significantly enriched.

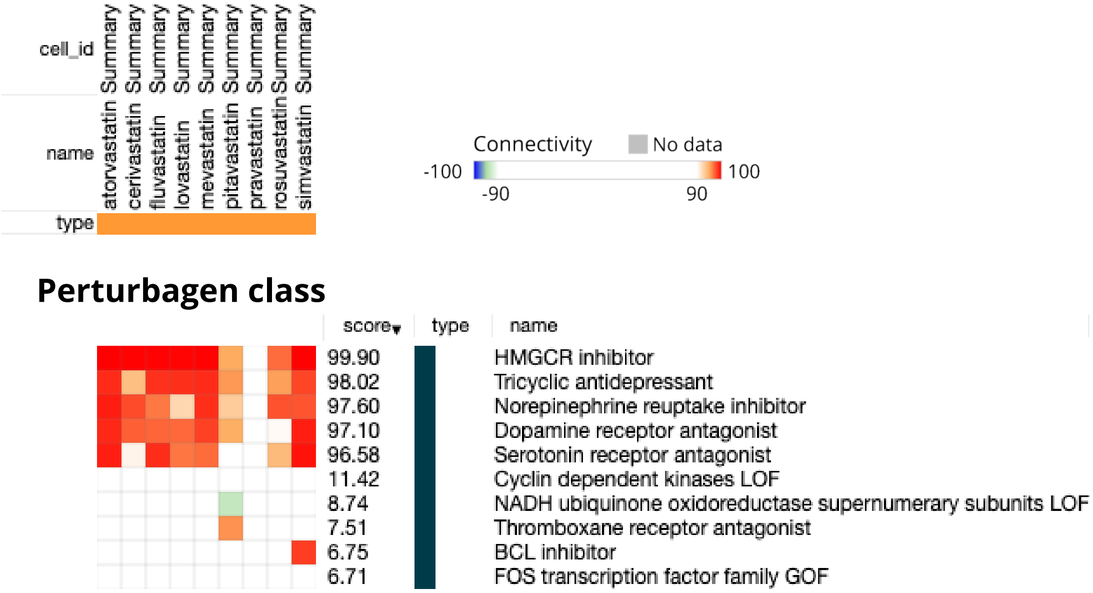

Supplementary Figure 7. Antidepressant classes ranked amongst top compound classes with highly similar gene expression signatures to statins. Preliminary query of the reference gene expression signatures in CMap database was performed by querying the connectivity profiles of "HMGCR inhibitors" using the online CLUE platform (https://clue.io) (accessed on September 9th, 2022) [2]. The top five compound (perturbagen) classes, identified to have the highest median connectivity score to statins, and the bottom five compound classes with the lowest median connectivity scores to statins, are shown. Compound classes are curated by CMap by grouping compounds that share the same mechanisms of action or biological functions, as identified by literature evidence. The median connectivity score (the "score" column) across the various statins are shown for each compound class. Heatmap shows the summary connectivity score (summarized across various cell lines) between each query statin compound (columns) and each compound class (rows). Image was adapted from the online CLUE platform.

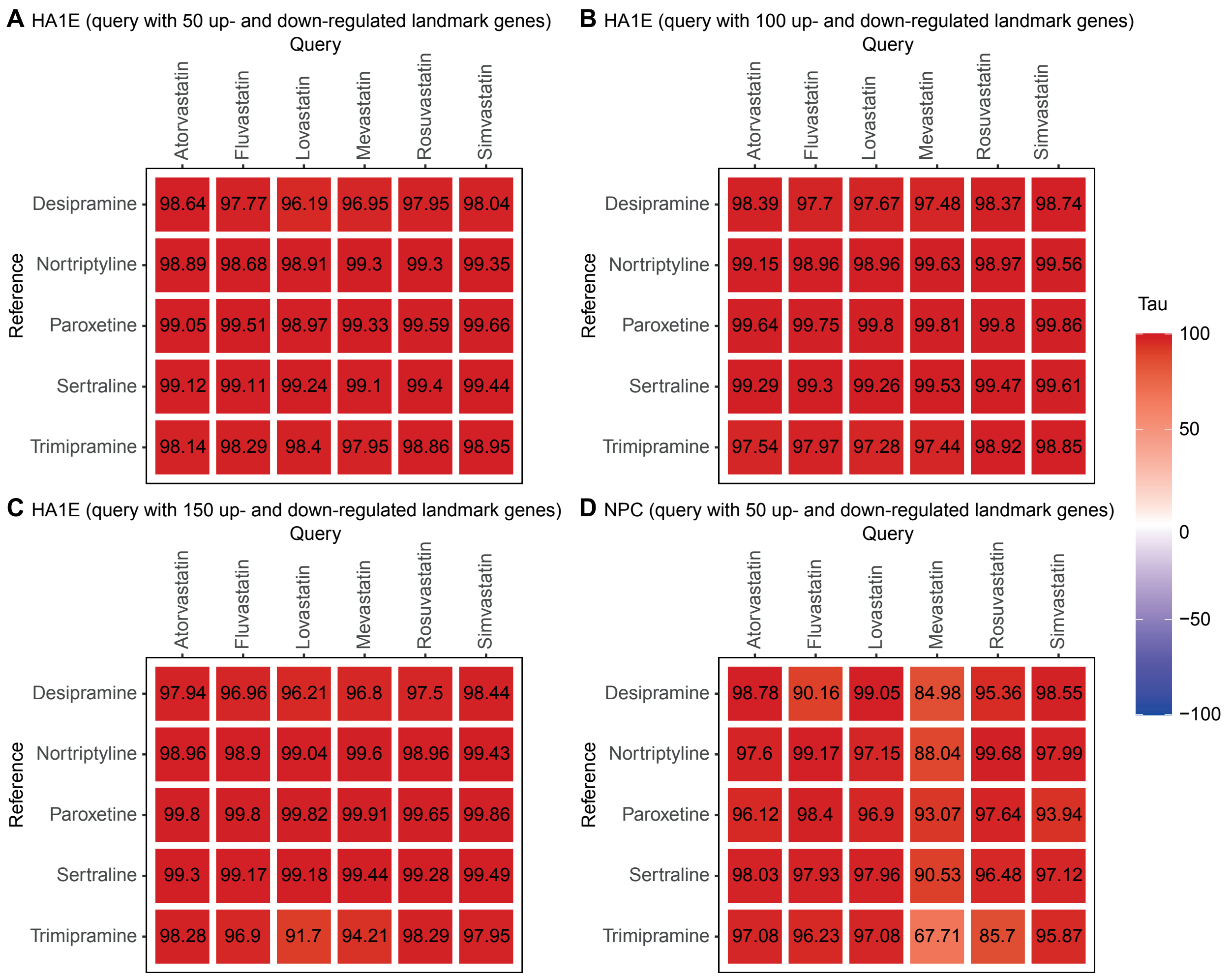

Supplementary Figure 8. Connectivity scores between query statin signatures and reference signatures of selected antidepressants. The connectivity scores shown here were generated by submitting statin query signatures to CLUE (https://clue.io). For statin gene expression signatures from HA1E cells, the connectivity profile was generated using (**A**) top 50, (**B**) top 100, and (**C**) top 150 up-regulated and down-regulated landmark genes. (**D**) For statin gene expression signatures from NPC cells, the connectivity profile was generated using the top 50 up-regulated and down-regulated landmark genes. Columns show the statin query signatures, and rows show the reference antidepressant signatures in the CMap database. The corresponding connectivity scores are annotated on the heatmap.

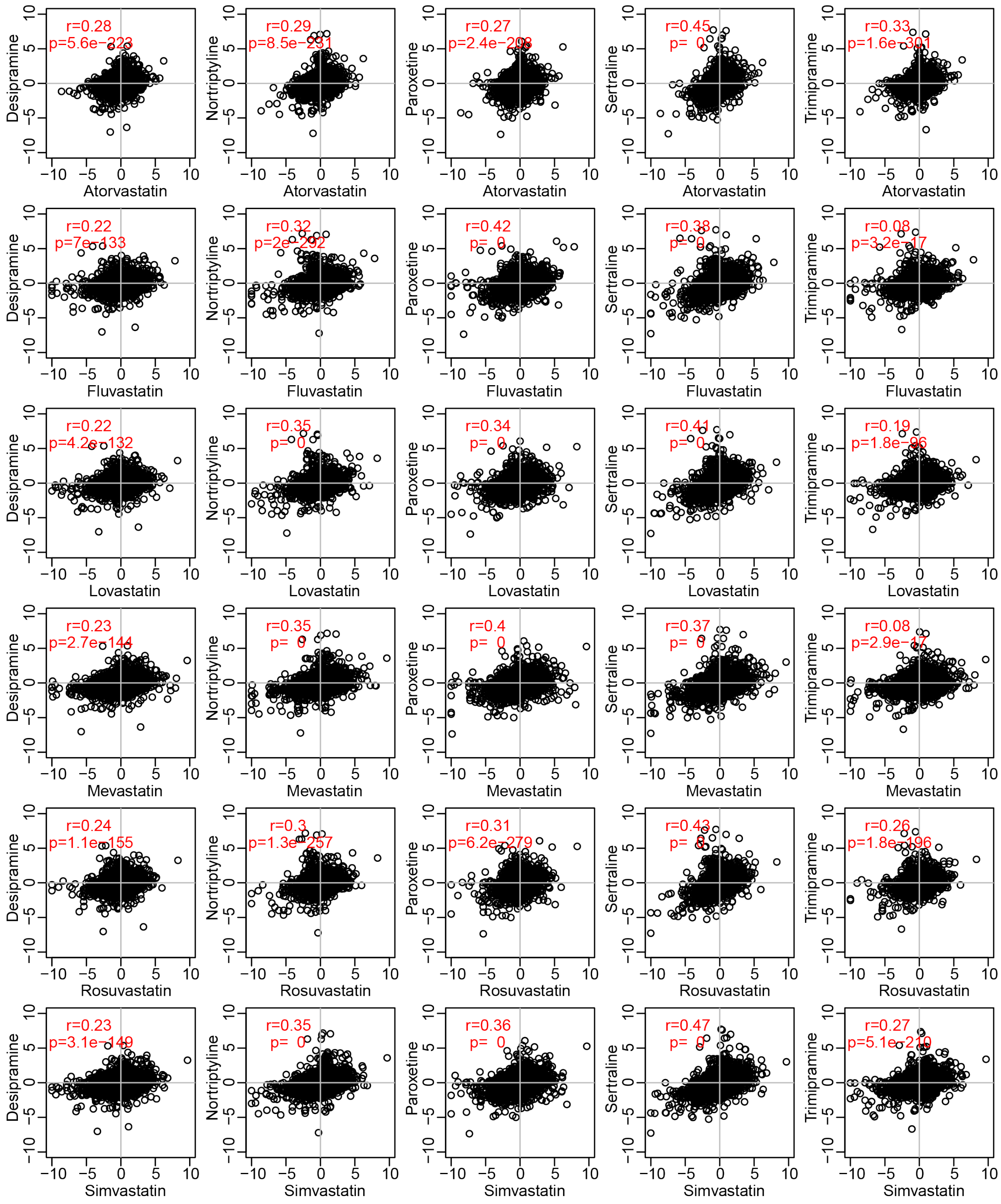

Supplementary Figure 9. Pairwise correlation of gene expression changes induced by statins and antidepressants in HA1E cells. The changes in gene expression (z-scores) of 12 328 genes profiled by CMap were compared for each pair of statin and antidepressant using Pearson correlation. The Pearson correlation coefficients (r) and the corresponding two-sided p-values (p) are shown.

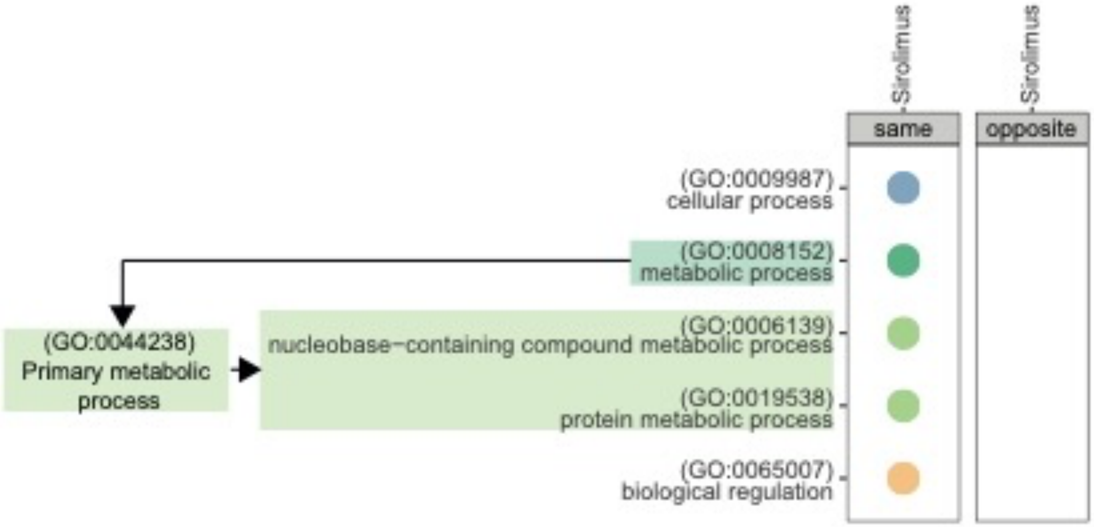

Supplementary Figure 10. Results of pathway enrichment analysis performed for genes perturbed by both statins and sirolimus. A total of 43 and 0 biological process terms were identified as significantly enriched amongst genes perturbed in the same and opposite directions, respectively, by statins and sirolimus. Y-axis shows the ancestor biological process terms, as well as the child terms of primary metabolic process (GO:0044238) (a child term of "metabolic process"). The arrows indicate ancestor-to-child relationships of GO terms. The bubble plots show the direction of gene perturbation for which the biological processes were identified as significantly enriched between at least one statin-sirolimus pair.

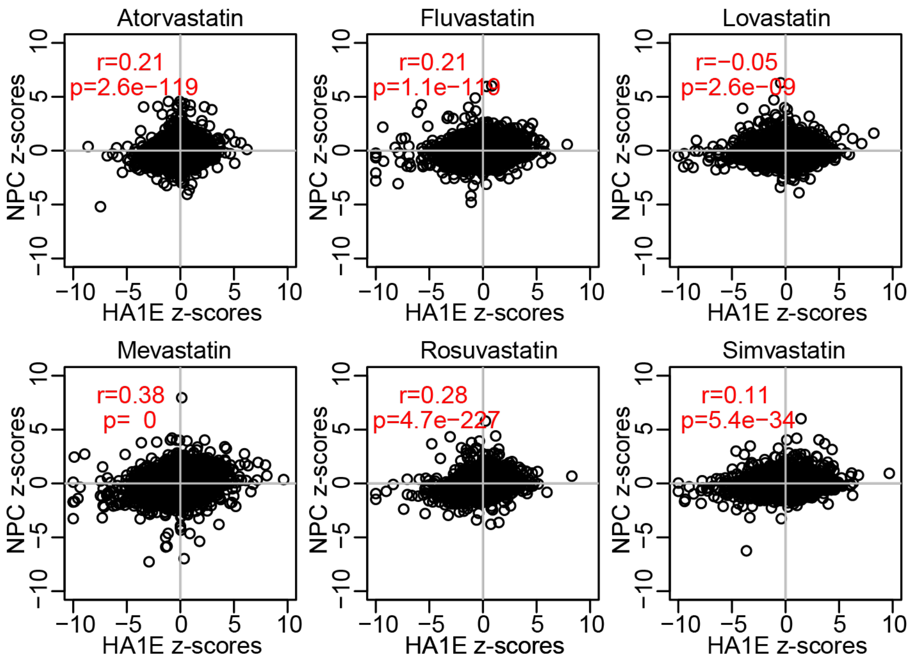

Supplementary Figure 11. Pairwise correlation of gene expression changes induced by statins in HA1E (x-axis) and NPC (y-axis) cells. Changes in gene expression (z-scores) of 12 328 genes profiled by CMap were compared for each statin using pairwise Pearson correlation. The Pearson correlation coefficients (r) and the corresponding two-sided p-values (p) are shown.

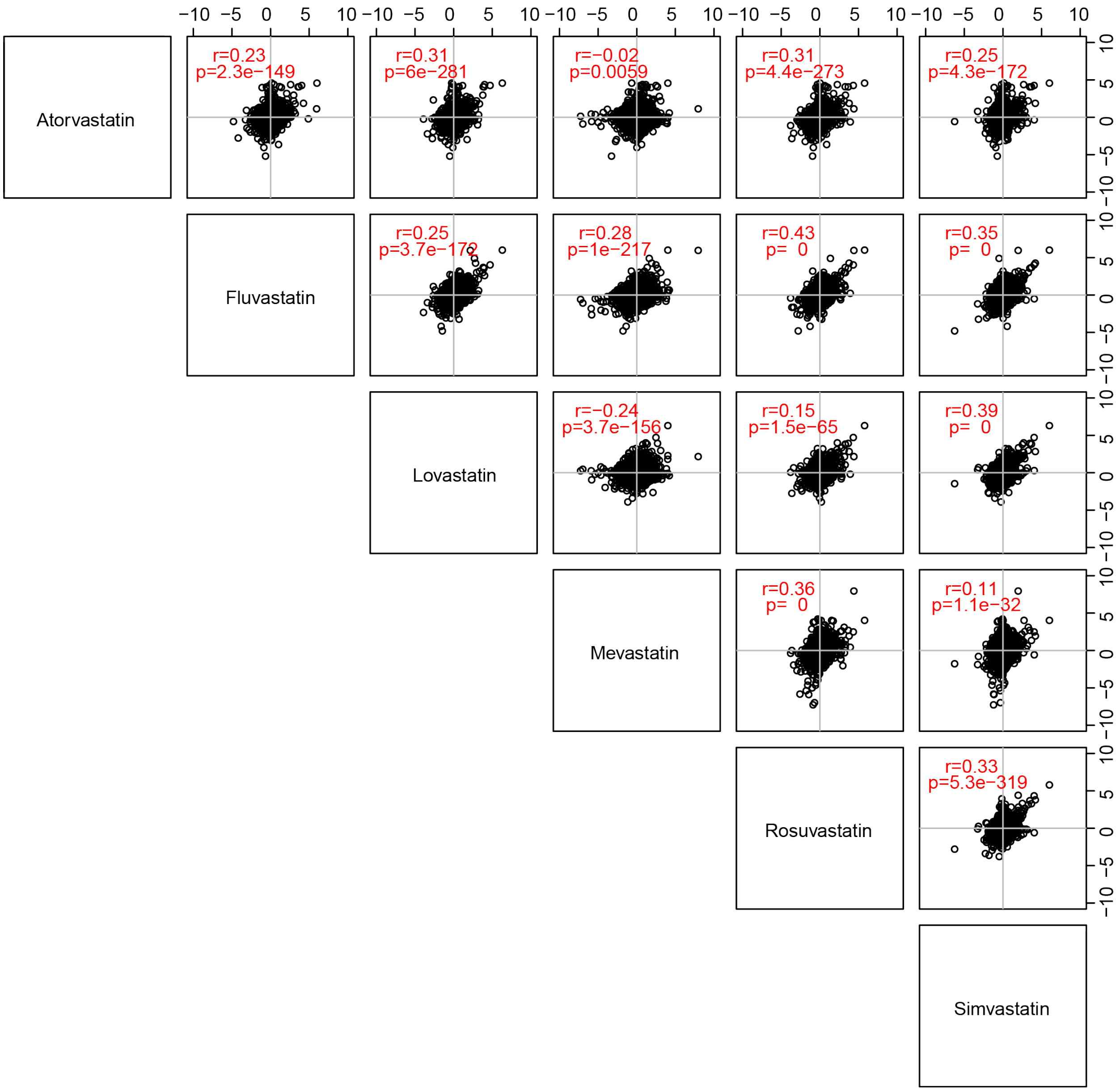

Supplementary Figure 12. Statins induced mostly concordant transcriptional responses in NPC cells. Changes in gene expression (z-scores) of 12 328 genes profiled by CMap were compared for each pair of statin compounds using Pearson correlation. The Pearson correlation coefficients (r) and the corresponding two-sided p-values (p) are shown.

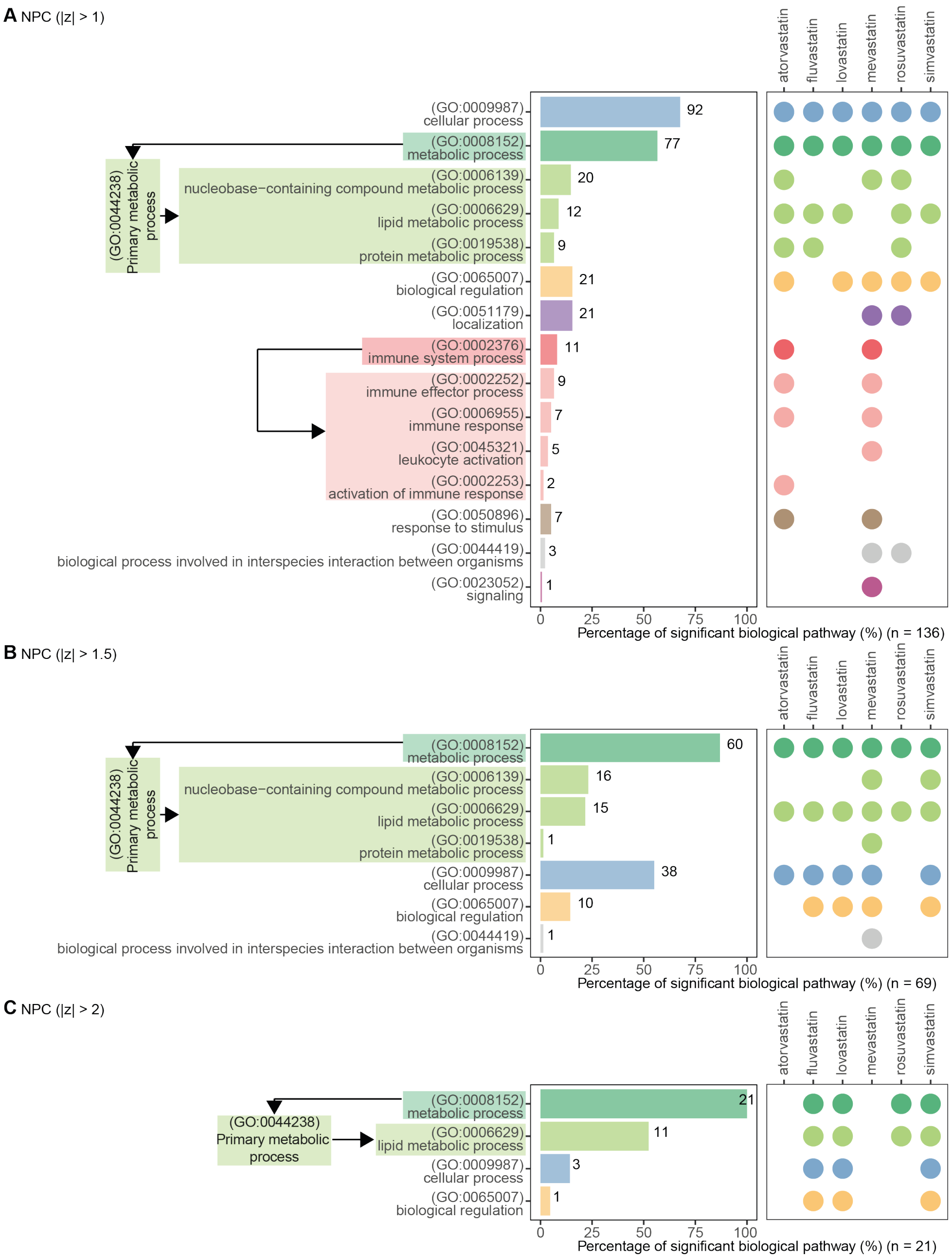

Supplementary Figure 13. Pathway enrichment results of statin-induced differentially expressed genes in NPC cells. Pathway enrichment analysis was performed for up- and down-regulated genes, defined by (**A**) absolute z-score > 1, (**B**) absolute z-score > 1.5 and (**C**) absolute z-score > 2, where a total of 136, 69 and 21 biological pathways, respectively, were identified as significantly enriched. Enriched biological process terms were categorized into high-level ancestor terms. Y-axis shows the ancestor biological process terms, as well as the child terms of primary metabolic process (a child term of "metabolic process") and immune system process. The arrows indicate ancestor-to-child relationships of GO terms. Bar graph shows the percentages of significant GO biological process terms annotated with each ancestor term, with the corresponding counts shown on the graph. The bubble plot shows the statin compounds for which the biological processes were identified as significantly enriched.

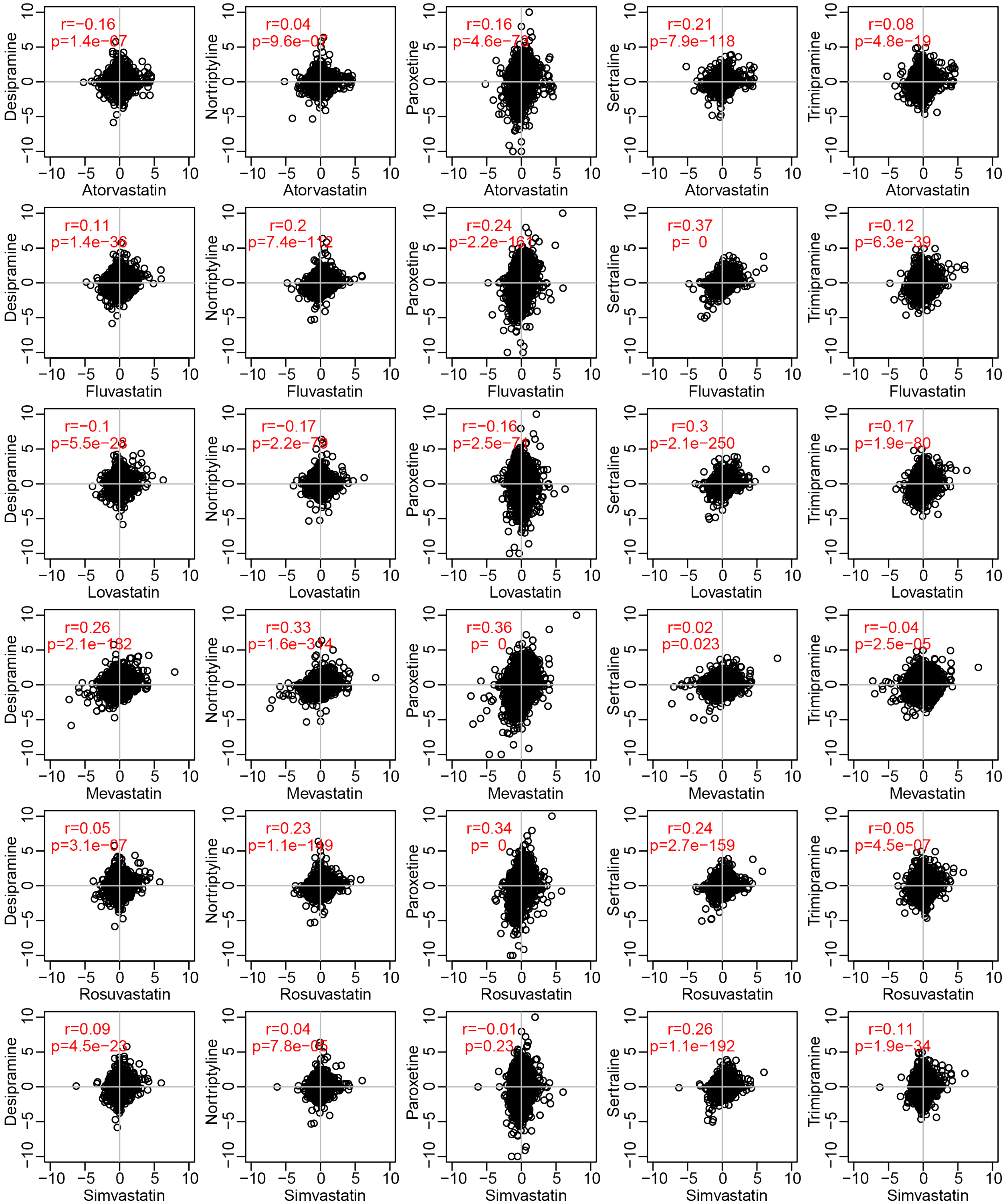

Supplementary Figure 14. Pairwise correlation of gene expression changes induced by statins and antidepressants in NPC cells. The changes in gene expression (z-scores) of 12 328 genes profiled by CMap were compared for each pair of statin and antidepressant using Pearson correlation. The Pearson correlation coefficients (r) and the corresponding two-sided p-values (p) are shown.

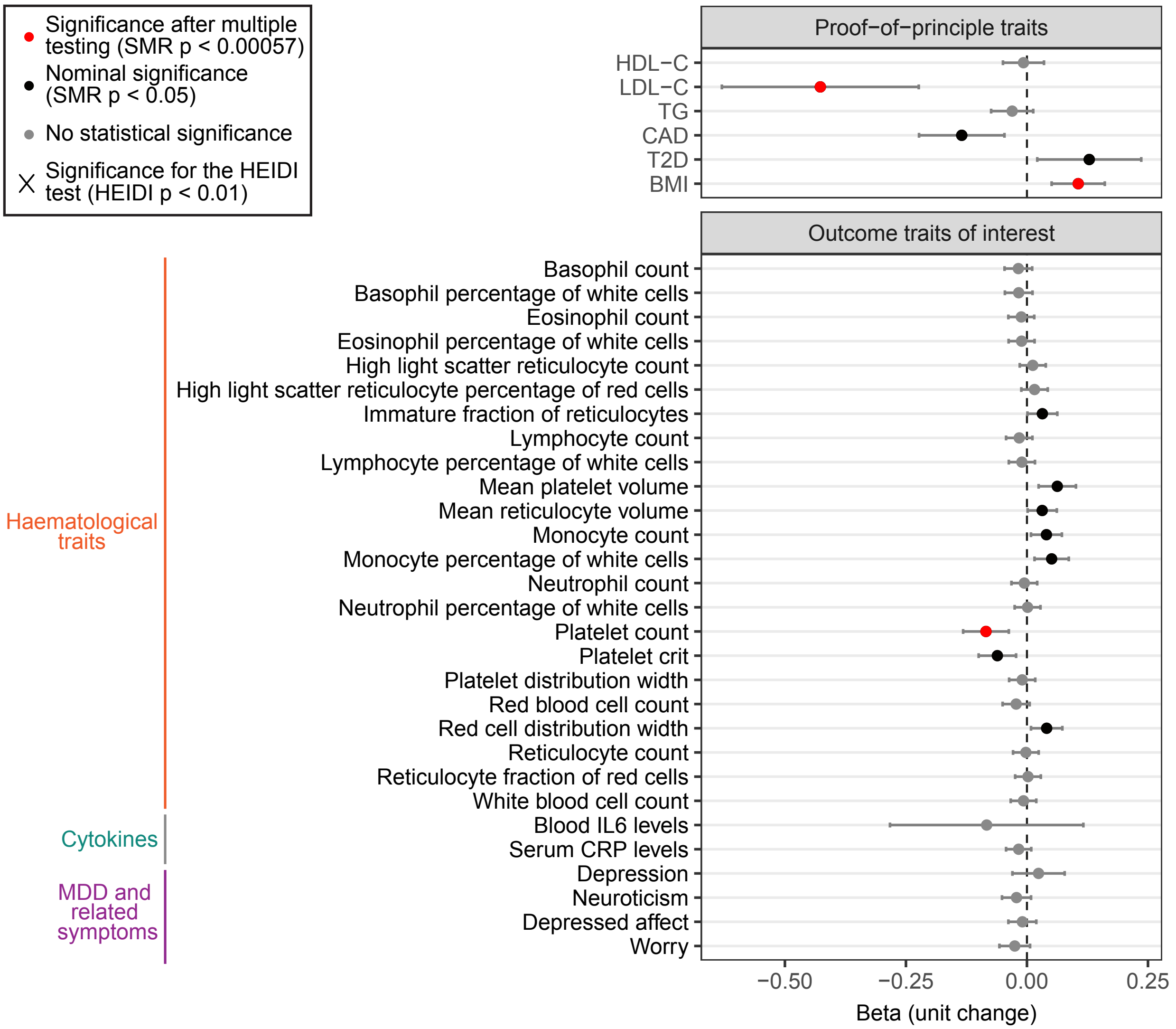

Supplementary Figure 15**.** MR analyses of *HMGCR* gene expression in the brain prefrontal cortex with various haematological, cytokine and depression-related traits. Dot plot shows the associations (beta) between gene expression and traits, and error bars show the 95% confidence intervals. The effect sizes are harmonized to represent the changes in trait per one standard deviation decrease in *HMGCR* expression (thus reflecting genetically proxied HMGCR inhibition). The units of beta values are not standardized. Red dots represent associations with statistical significance after multiple testing correction ($p < 0.00057$), and black dots represent associations with nominal statistical significance ($p < 0.05$). Associations with significant HEIDI p-values are marked by ×.

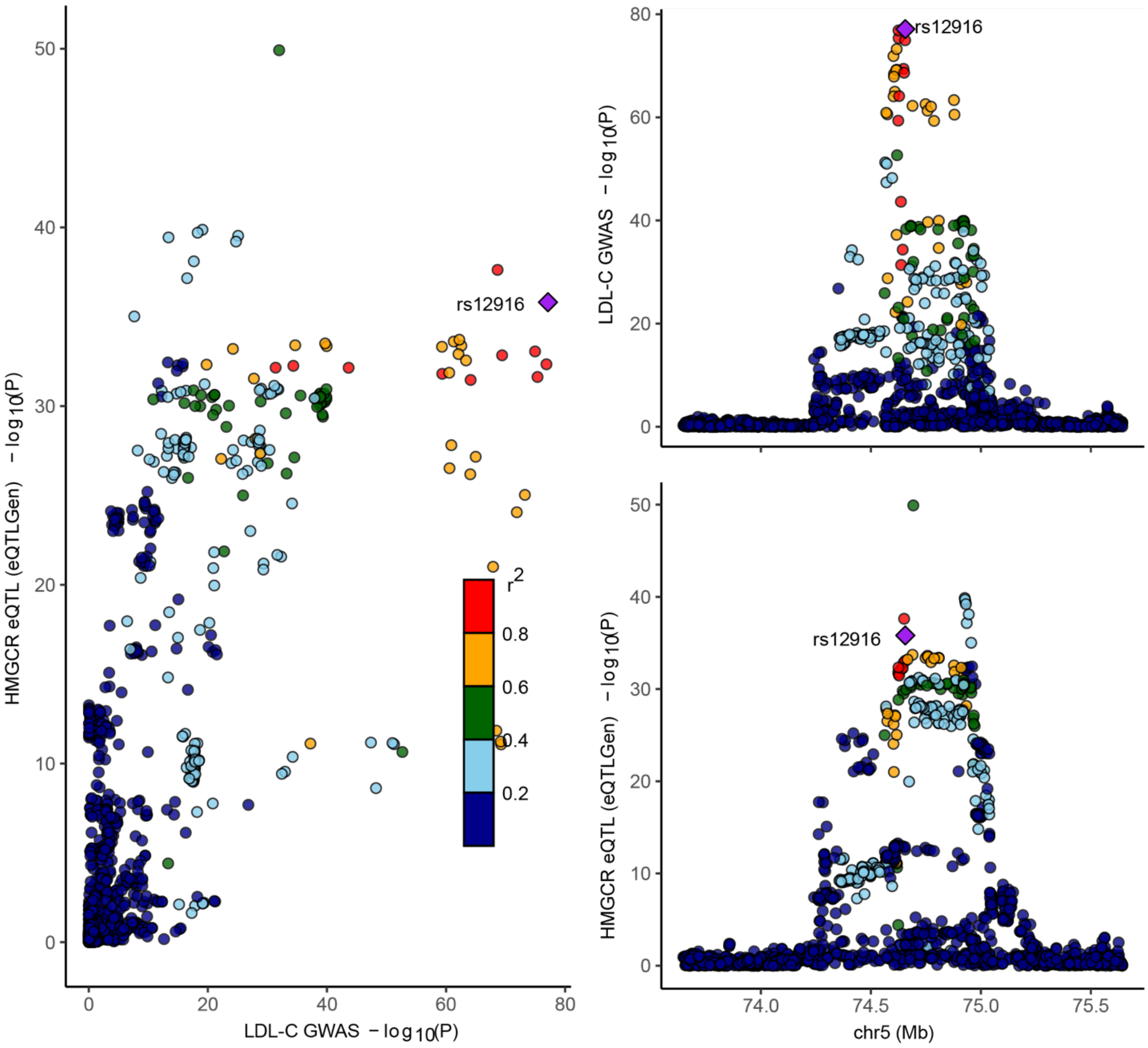

Supplementary Figure 16. LocusCompare plot of *HMGCR* eQTLs (eQTLGen) and LDL-C GWAS summary statistics. Each dot represents a SNP and is colored based on their degree of LD (r^2) with rs12916 (purple diamond). The LocusZoom plots (right panels) show the significance ($-\log_{10}P$) of SNPs against their genomic locations. The scatter plot (left panel) shows the SNP significance in the eQTL dataset plotted against the SNP significance in the LDL-C GWAS dataset.

Supplementary Tables

Supplementary Table 1. The CMap signatures selected for analyses

Compound	Cell line	CMap signature
Atorvastatin	HA1E	CPC006_HA1E_24H:BRD-U88459701-000-01-8:10
Fluvastatin	HA1E	CPC004_HA1E_24H:BRD-K66296774-001-02-0:10
Lovastatin	HA1E	CPC003_HA1E_24H:BRD-K09416995-001-21-7:10
Mevastatin	HA1E	CPC001_HA1E_24H:BRD-K94441233-001-03-1:10
Pravastatin	HA1E	CPC002_HA1E_24H:BRD-K60511616-236-01-4:10
Rosuvastatin	HA1E	CPC004_HA1E_24H:BRD-K82941592-238-02-9:10
Simvastatin	HA1E	CPC005_HA1E_24H:BRD-A81772229-001-01-6:10
Desipramine	HA1E	CPC004_HA1E_24H:BRD-K60762818-003-15-3:10
Nortriptyline	HA1E	CPC002_HA1E_24H:BRD-K91263825-001-03-6:10
Paroxetine	HA1E	CPC004_HA1E_24H:BRD-K37991163-003-06-8:10
Sertraline	HA1E	CPC004_HA1E_24H:BRD-K82036761-003-07-0:10
Trimipramine	HA1E	CPC004_HA1E_24H:BRD-A19195498-050-09-1:10
Alvespimycin	HA1E	CPC014_HA1E_6H:BRD-K83988098-003-01-8:10
Sirolimus	HA1E	CPC012_HA1E_6H:BRD-K89626439-001-01-0:10
Atorvastatin	NPC	NMH001_NPC_24H:BRD-K69726342-001-02-6:10
Fluvastatin	NPC	CPC015_NPC_24H:BRD-K66296774-001-02-0:10
Lovastatin	NPC	CPC015_NPC_24H:BRD-K09416995-001-21-7:10
Mevastatin	NPC	CPC016_NPC_24H:BRD-K94441233-001-03-1:10
Rosuvastatin	NPC	CPC015_NPC_24H:BRD-K82941592-238-02-9:10
Simvastatin	NPC	CPC015_NPC_24H:BRD-K22134346-001-11-6:10
Desipramine	NPC	CPC017_NPC_24H:BRD-K60762818-003-15-3:10
Nortriptyline	NPC	CPC018_NPC_24H:BRD-K91263825-001-03-6:10
Paroxetine	NPC	CPC015_NPC_24H:BRD-K37991163-003-06-8:10
Sertraline	NPC	CPC015_NPC_24H:BRD-K82036761-003-07-0:10
Trimipramine	NPC	CPC016_NPC_24H:BRD-A19195498-050-09-1:10

Supplementary Table 2. Number of up-regulated and down-regulated genes upon statin exposure in HA1E cells, defined using different z-score thresholds

Statin	Z > 1	Z < -1	Z > 1.5	Z < -1.5	Z > 2	Z < -2
Atorvastatin	1597	1606	677	685	224	297
Fluvastatin	4252	1599	2431	919	1017	520
Lovastatin	2508	1777	1134	965	457	515
Mevastatin	4750	1874	2890	1141	1435	697
Rosuvastatin	2034	1502	851	723	318	345
Simvastatin	2701	1949	1436	1129	664	658

Supplementary Table 3. Antidepressants profiled in the CMap database

ATC class	ATC code	Drug name	CMap ID
Non-selective monoamine reuptake inhibitors (N06AA) (tricyclic antidepressant)	N06AA01	Desipramine	BRD-K60762818
	N06AA02	Imipramine	BRD-K38436528
	N06AA04	Clomipramine	BRD-K52989797
	N06AA06	Trimipramine	BRD-A19195498
	N06AA07	Lofepramine	BRD-K82147103
	N06AA08	Dibenzepin	BRD-K79145749
	N06AA09	Amitriptyline	BRD-K53737926
	N06AA10	Nortriptyline	BRD-K91263825
	N06AA11	Protriptyline	BRD-K42098891
	N06AA12	Doxepin	BRD-K36616567
	N06AA16	Dosulepin	BRD-K54759182
	N06AA17	Amoxapine	BRD-K02265150
	N06AA21	Maprotiline	BRD-K03319035
Selective serotonin reuptake inhibitors (N06AB)	N06AB03	Fluoxetine	BRD-A31159102
	N06AB04	Citalopram	BRD-A47598013
	N06AB05	Paroxetine	BRD-K37991163
	N06AB06	Sertraline	BRD-K82036761
	N06AB07	Alaproclate	BRD-A14966924
	N06AB08	Fluvoxamine	BRD-K72676686
	N06AB10	Escitalopram	BRD-K70301876
Monoamine oxidase inhibitors, non-selective (N06AF)	N06AF01	Isocarboxazid	BRD-K93332168
	N06AF02	Nialamide	BRD-K12102668
	N06AF03	Phenelzine	BRD-K87024524
	N06AF04	Tranylcypromine	BRD-A43974575
Monoamine oxidase A inhibitors (N06AG)	N06AG02	Moclobemide	BRD-K07237224
Other antidepressants (N06AX)	N06AX03	Mianserin	BRD-A19661776
	N06AX04	Nomifensine	BRD-A29644307
	N06AX05	Trazodone	BRD-K70778732
	N06AX06	Nefazodone	BRD-K90789829
	N06AX07	Minaprine	BRD-K02867583
	N06AX08	Bifemelane	BRD-K18779551
	N06AX11	Mirtazapine	BRD-A64977602
	N06AX12	Bupropion	BRD-A05186015
	N06AX14	Tianeptine	BRD-A53077924
	N06AX16	Venlafaxine	BRD-A51714012
	N06AX17	Milnacipran	BRD-K02227374
	N06AX18	Reboxetine	BRD-A43974499
	N06AX21	Duloxetine	BRD-K71103788

Supplementary Table 4. Sources of eQTL datasets

Dataset	Tissue used in the study	Sample size	Ancestry	Reference
eQTLGen	Blood	31684	Predominantly European	Võsa et al. [23]
GTEx (version 8)	Whole blood	670	Predominantly European	Aguet et al. [38]
PsychENCODE	Brain prefrontal cortex	1387	Predominantly European	Gandal et al. [24]

Supplementary Table 5. Sources of GWAS summary statistics

Trait and sample size	Source	Access date
Haematological parameters (408112)<br>• Basophil count<br>• Basophil percentage of white cells<br>• Eosinophil count<br>• Eosinophil percentage of white cells<br>• High light scatter reticulocyte count<br>• High light scatter reticulocyte percentage of red cells<br>• Immature fraction of reticulocytes<br>• Lymphocyte count<br>• Lymphocyte percentage of white cells<br>• Mean platelet volume<br>• Mean reticulocyte volume<br>• Monocyte count<br>• Monocyte percentage of white cells<br>• Neutrophil count<br>• Neutrophil percentage of white cells<br>• Platelet count<br>• Platelet crit<br>• Platelet distribution width<br>• Red blood cell count<br>• Red cell distribution width<br>• Reticulocyte count<br>• Reticulocyte fraction of red cells<br>• White blood cell count	Vuckovic et al. [42]	May 10th, 2022
Blood IL6 level (8189)	Ahola-Olli et al. [43]	September 14th, 2021
Serum CRP level (418642)	Han et al. [44]<br>(GWAS catalogue ID: GCST009777)	September 21st, 2021
Lipids<br>• HDL-C (187167)<br>• LDL-C (173082)<br>• TG (177861)	Willer et al. [45]	September 6th, 2021
CAD (34541 cases and 261984 controls from the UK Biobank cohort)	van der Harst and Verweij [46]<br>(openGWAS ID: ebi-a-GCST005194)	September 9th, 2021
T2D (62892 cases and 596424 controls)	Xue et al. [47]<br>(openGWAS ID: ebi-a-GCST006867)	September 9th, 2021
BMI (461460)	Hemani et al. [48]<br>(openGWAS ID: ukb-b-19953)	September 10th, 2021
Depression (170756 cases and 329443 controls) (excluding 23andMe data)	Howard et al. [49]	February 15th, 2022
Neuroticism (380060)	Nagel et al. [50]	September 22nd, 2021
Depressed affect (357957)<br>Worrying (348219)	Nagel et al. [51]	September 22nd, 2021<br>September 22nd, 2021

Supplementary Table 6. Chi-square analysis of antidepressant enrichment amongst compounds showing high connectivity scores to statins (Tau > 90)

Cell line	Number of up- and down-regulated landmark genes used to generate connectivity profile	Compounds with average Tau > 90		Compounds with average Tau ≤ 90		Chi-square p-value with Yates' correction
		Total	Antidepressants (% of total)	Total	Antidepressants (% of total)	
HA1E	50	188	12 (6.38%)	2305	26 (1.13%)	9E-08
HA1E	100	191	10 (5.24%)	2302	28 (1.22%)	5.1E-05
HA1E	150	179	9 (5.03%)	2314	29 (1.25%)	0.00026
NPC	50	121	6 (4.96%)	2424	32 (1.34%)	0.0046

References

1. Lamb J, Crawford ED, Peck D, Modell JW, Blat IC, Wrobel MJ, et al. The Connectivity Map: using gene-expression signatures to connect small molecules, genes, and disease. Science. 2006;313(5795):1929-35.

2. Subramanian A, Narayan R, Corsello SM, Peck DD, Natoli TE, Lu X, et al. A next generation connectivity map: L1000 platform and the first 1,000,000 profiles. Cell. 2017;171(6):1437-52.e17.

3. Uhlén M, Fagerberg L, Hallström Björn M, Lindskog C, Oksvold P, Mardinoglu A, et al. Tissue-based map of the human proteome. Science. 2015;347(6220):1260419.

4. Broad DepMap. DepMap 21Q4 Public. figshare. Dataset.2021.

5. Hu Y, Bobb D, He J, Hill DA, Dome JS. The HSP90 inhibitor alvespimycin enhances the potency of telomerase inhibition by imetelstat in human osteosarcoma. Cancer Biol Ther. 2015;16(6):949-57.

6. Sehgal SN. Sirolimus: its discovery, biological properties, and mechanism of action. Transplant Proc. 2003;35(3, Supplement):S7-S14.

7. Morrisett JD, Abdel-Fattah G, Hoogeveen R, Mitchell E, Ballantyne CM, Pownall HJ, et al. Effects of sirolimus on plasma lipids, lipoprotein levels, and fatty acid metabolism in renal transplant patients. J Lipid Res. 2002;43(8):1170-80.

8. Ma KL, Ruan XZ, Powis SH, Moorhead JF, Varghese Z. Anti-atherosclerotic effects of sirolimus on human vascular smooth muscle cells. Am J Physiol Heart Circ Physiol. 2007;292(6):H2721-H8.

9. Shi Z, Geng Y, Liu J, Zhang H, Zhou L, Lin Q, et al. Single-cell transcriptomics reveals gene signatures and alterations associated with aging in distinct neural stem/progenitor cell subpopulations. Protein & Cell. 2018;9(4):351-64.

10. Ashburner M, Ball CA, Blake JA, Botstein D, Butler H, Cherry JM, et al. Gene Ontology: tool for the unification of biology. Nat Genet. 2000;25(1):25-9.

11. Carlson M. GO.db: A set of annotation maps describing the entire Gene Ontology. R package version 3.8.2. 2019.

12. WHO Collaborating Centre for Drug Statistics Methodology. Guidelines for ATC classification and DDD assignment, 2022. In: World Health Organization, editor. Oslo2021.

13. Davey Smith G. Capitalizing on Mendelian randomization to assess the effects of treatments. J R Soc Med. 2007;100(9):432-5.

14. Davey Smith G, Lawlor DA, Harbord R, Timpson N, Day I, Ebrahim S. Clustered environments and randomized genes: a fundamental distinction between conventional and genetic eidemiology. PLoS Med. 2007;4(12):e352.

15. Palmer TM, Nordestgaard BG, Benn M, Tybjærg-Hansen A, Davey Smith G, Lawlor DA, et al. Association of plasma uric acid with ischaemic heart disease and blood pressure: mendelian randomisation analysis of two large cohorts. BMJ 2013;347:f4262.

16. Davey Smith G, Ebrahim S. 'Mendelian randomization': can genetic epidemiology contribute to understanding environmental determinants of disease? Int J Epidemiol. 2003;32(1):1-22.

17. Davies NM, Holmes MV, Davey Smith G. Reading Mendelian randomisation studies: a guide, glossary, and checklist for clinicians. BMJ. 2018;362:k601.

18. Swerdlow DI, Kuchenbaecker KB, Shah S, Sofat R, Holmes MV, White J, et al. Selecting instruments for Mendelian randomization in the wake of genome-wide association studies. Int J Epidemiol. 2016;45(5):1600-16.

19. Porcu E, Rüeger S, Lepik K, Agbessi M, Ahsan H, Alves I, et al. Mendelian randomization integrating GWAS and eQTL data reveals genetic determinants of complex and clinical traits. Nat Commun. 2019;10(1):3300.

20. Nicolae DL, Gamazon E, Zhang W, Duan S, Dolan ME, Cox NJ. Trait-associated SNPs are more likely to be eQTLs: annotation to enhance discovery from GWAS. PLoS Genet. 2010;6(4):e1000888.

21. Chauquet S, Zhu Z, O'Donovan MC, Walters JTR, Wray NR, Shah S. Association of antihypertensive drug target genes with psychiatric disorders: a Mendelian randomization study. JAMA Psychiatry. 2021;78(6):623-31.

22. Baird DA, Liu JZ, Zheng J, Sieberts SK, Perumal T, Elsworth B, et al. Identifying drug targets for neurological and psychiatric disease via genetics and the brain transcriptome. PLoS Genet. 2021;17(1):e1009224.

23. Võsa U, Claringbould A, Westra H-J, Bonder MJ, Deelen P, Zeng B, et al. Large-scale cis- and trans-eQTL analyses identify thousands of genetic loci and polygenic scores that regulate blood gene expression. Nat Genet. 2021;53(9):1300-10.

24. Gandal MJ, Zhang P, Hadjimichael E, Walker Rebecca L, Chen C, Liu S, et al. Transcriptome-wide isoform-level dysregulation in ASD, schizophrenia, and bipolar disorder. Science. 2018;362(6420):eaat8127.

25. Würtz P, Wang Q, Soininen P, Kangas AJ, Fatemifar G, Tynkkynen T, et al. Metabolomic profiling of statin use and genetic inhibition of HMG-CoA reductase. J Am Coll Cardiol. 2016;67(10):1200-10.

26. Swerdlow DI, Preiss D, Kuchenbaecker KB, Holmes MV, Engmann JE, Shah T, et al. HMG-coenzyme A reductase inhibition, type 2 diabetes, and bodyweight: evidence from genetic analysis and randomised trials. The Lancet. 2015;385(9965):351-61.

27. Schooling CM, Zhao JV, Au Yeung SL, Leung GM. Investigating pleiotropic effects of statins on ischemic heart disease in the UK Biobank using Mendelian randomisation. Elife. 2020;9:e58567.

28. Li S, Schooling CM. A phenome-wide association study of genetically mimicked statins. BMC Med. 2021;19(1):151.

29. Wishart DS, Knox C, Guo AC, Shrivastava S, Hassanali M, Stothard P, et al. DrugBank: a comprehensive resource for in silico drug discovery and exploration. Nucleic Acids Res. 2006;34(suppl_1):D668-D72.

30. Hynes RO. Integrins: Versatility, modulation, and signaling in cell adhesion. Cell. 1992;69(1):11-25.

31. Keum S, Lee HK, Chu P-L, Kan MJ, Huang M-N, Gallione CJ, et al. Natural genetic variation of Integrin Alpha L (Itgal) modulates ischemic brain injury in stroke. PLoS Genet. 2013;9(10):e1003807.

32. Kallen J, Welzenbach K, Ramage P, Geyl D, Kriwacki R, Legge G, et al. Structural basis for LFA-1 inhibition upon lovastatin binding to the CD11a I-domain. J Mol Biol. 1999;292(1):1-9.

33. Weitz-Schmidt G, Welzenbach K, Brinkmann V, Kamata T, Kallen J, Bruns C, et al. Statins selectively inhibit leukocyte function antigen-1 by binding to a novel regulatory integrin site. Nat Med. 2001;7(6):687-92.

34. Wang Y, Li D, Jones D, Bassett R, Sale GE, Khalili J, et al. Blocking LFA-1 activation with lovastatin prevents graft-versus-host disease in mouse bone marrow transplantation. Biol Blood Marrow Transplant. 2009;15(12):1513-22.

35. Lin Y-C, Lin J-H, Chou C-W, Chang Y-F, Yeh S-H, Chen C-C. Statins increase p21 through inhibition of histone deacetylase activity and release of promoter-associated HDAC1/2. Cancer Res. 2008;68(7):2375.

36. Jenuwein T, Allis CD. Translating the histone code. Science. 2001;293(5532):1074-80.

37. Roth EM, Davidson MH. PCSK9 inhibitors: mechanism of action, efficacy, and safety. Rev Cardiovasc Med. 2018;19(S1):S31-s46.

38. Aguet F, Anand S, Ardlie Kristin G, Gabriel S, Getz Gad A, Graubert A, et al. The GTEx Consortium atlas of genetic regulatory effects across human tissues. Science. 2020;369(6509):1318-30.

39. Zhang J, Dutta D, Köttgen A, Tin A, Schlosser P, Grams ME, et al. Plasma proteome analyses in individuals of European and African ancestry identify cis-pQTLs and models for proteome-wide association studies. Nat Genet. 2022;54(5):593-602.

40. Zhu Z, Zhang F, Hu H, Bakshi A, Robinson MR, Powell JE, et al. Integration of summary data from GWAS and eQTL studies predicts complex trait gene targets. Nat Genet. 2016;48(5):481-7.

41. Liu B, Gloudemans MJ, Rao AS, Ingelsson E, Montgomery SB. Abundant associations with gene expression complicate GWAS follow-up. Nat Genet. 2019;51(5):768-9.

42. Vuckovic D, Bao EL, Akbari P, Lareau CA, Mousas A, Jiang T, et al. The polygenic and monogenic basis of blood traits and diseases. Cell. 2020;182(5):1214-31.e11.

43. Ahola-Olli AV, Würtz P, Havulinna AS, Aalto K, Pitkänen N, Lehtimäki T, et al. Genome-wide association study identifies 27 loci influencing concentrations of circulating cytokines and growth factors. Am J Hum Genet. 2017;100(1):40-50.

44. Han X, Ong J-S, An J, Hewitt AW, Gharahkhani P, MacGregor S. Using Mendelian randomization to evaluate the causal relationship between serum C-reactive protein levels and age-related macular degeneration. Eur J Epidemiol. 2020;35(2):139-46.

45. Willer CJ, Schmidt EM, Sengupta S, Peloso GM, Gustafsson S, Kanoni S, et al. Discovery and refinement of loci associated with lipid levels. Nat Genet. 2013;45(11):1274-83.

46. van der Harst P, Verweij N. Identification of 64 novel genetic loci provides an expanded view on the genetic architecture of coronary artery disease. Circ Res. 2018;122(3):433-43.

47. Xue A, Wu Y, Zhu Z, Zhang F, Kemper KE, Zheng Z, et al. Genome-wide association analyses identify 143 risk variants and putative regulatory mechanisms for type 2 diabetes. Nat Commun. 2018;9(1):2941.

48. Hemani G, Zheng J, Elsworth B, Wade KH, Haberland V, Baird D, et al. The MR-Base platform supports systematic causal inference across the human phenome. Elife. 2018;7:e34408.

49. Howard DM, Adams MJ, Clarke TK, Hafferty JD, Gibson J, Shirali M, et al. Genome-wide meta-analysis of depression identifies 102 independent variants and highlights the importance of the prefrontal brain regions. Nat Neurosci. 2019;22(3):343-52.

50. Nagel M, Watanabe K, Stringer S, Posthuma D, van der Sluis S. Item-level analyses reveal genetic heterogeneity in neuroticism. Nat Commun. 2018;9(1):905.

51. Nagel M, Jansen PR, Stringer S, Watanabe K, de Leeuw CA, Bryois J, et al. Meta-analysis of genome-wide association studies for neuroticism in 449,484 individuals identifies novel genetic loci and pathways. Nat Genet. 2018;50(7):920-7.
